# Linc00312 Single Nucleotide Polymorphism as Biomarker for Chemoradiotherapy Induced Hematotoxicity in Nasopharyngeal Carcinoma Patients

**DOI:** 10.1155/2022/6707821

**Published:** 2022-08-08

**Authors:** Zhen Guo, Ya-Jing Wang, Bin-Sheng He, Ji Zhou

**Affiliations:** ^1^Academician Workstation, Changsha Medical University, Changsha 410219, China; ^2^Wuzhou Medical College, Wuzhou 543000, China; ^3^Hunan Key Laboratory of the Research and Development of Novel Pharmaceutical Preparations, Changsha Medical University, Changsha 410219, China

## Abstract

**Background:**

Linc00312 is downregulated in nasopharyngeal carcinoma (NPC) and associates with poor treatment efficacy. Genetic variations are the main cause of individual differences in treatment response. The objective of this study is to explore the relationship between genetic variations of linc00312 and the risk of chemoradiotherapy induced toxic reactions in NPC patients.

**Methods:**

We used a bioinformatics approach to select 3 single nucleotide polymorphisms (SNPs) with regulatory feature in linc00312 (rs12497104, rs15734, and rs164966). 505 NPC patients receiving chemoradiotherapy with complete follow-up data were recruited. Genotyping was carried out by MassARRAY iPLEX platform. Univariate logistic and multivariate logistic regression were used to analyze the risk factors responsible for toxic reactions of NPC patients.

**Results:**

Our result demonstrated that linc00312 rs15734 (*G* > *A*) was significantly associated with severe leukopenia in NPC patients underwent chemoradiotherapy (AA vs. GG, OR = 3.145, *P* = 0.029). In addition, the risk of severe leukopenia was remarkably increased to 5.635 times (*P* = 0.034) in female with rs15734 AA genotype compared to male with rs15734 GG genotype. Moreover, patients with rs12497104 (*G* > *A*) AA genotype showed a 67.5% lower risk of thrombocytopenia than those with GG genotype (*P* = 0.030). Remarkably, the younger patients (age < 47) with rs12497104 AA genotype displayed a 90% decreased risk of thrombocytopenia compared with older patients (age ≥ 47) carrying rs12497104 GG genotype (*P* = 0.030).

**Conclusions:**

Genetic variations of linc00312 affect the risk of chemoradiotherapy induced hematotoxicity in nasopharyngeal carcinoma patients and may serve as biomarkers for personalized medicine.

## 1. Introduction

Nasopharyngeal carcinoma (NPC) is a malignant tumor with distinct geographical and ethnic distribution, which is prevalent in Southeast Asia, North Africa, and Southern China. About 50% of the new NPC cases occur in China every year, causing a serious healthcare problem in China [1]. Radiotherapy and chemotherapy are the main treatment methods for NPC. Platinum-based concurrent chemoradiotherapy combined with induction chemotherapy or adjuvant chemotherapy has been identified as the standard protocol for locally advanced nasopharyngeal carcinoma [1]. Although the combination of radiotherapy and chemotherapy improves the killing effect on tumor cells, it also causes certain damage to normal cells, such as myelosuppression, radiation dermatitis, oral mucositis, and other toxic effects [2]. These toxic effects not only cause pain and discomfort for NPC patients but also lead to the interruption of treatment, affecting the final treatment effect.

It is not unusual to see that different patients may present with different degrees of toxic reactions despite receiving identical treatment regimen. This individual difference may be due to personal physical fitness and living habits, but recent studies have shown that human genome variation is the key factor responsible for individual differences in drug response [3]. Evolution of personalized medicine is widely acknowledged as the only way to overcome the inherent limitations of chemotherapy [4].

lncRNA is a kind of RNA with a length of more than 200 nt and does not have protein coding ability. It can widely participate in the regulation of various cellular biological processes at the transcription level, posttranscription level, and epigenetic level [5–8]. GWAS study found that only 7% of the disease-related loci were located in the protein coding region, and the remaining 93% were located in the noncoding region, suggesting that genetic variation in the noncoding region may play an important role in regulating the disease course [9]. Increasing evidence have shown that genetic variation of lncRNA is not only a risk site for cancer susceptibility but also plays an important role in drug response [10, 11].

Linc00312 is located at the 3p25.3 region of human chromosome. There are a lot of deletions in 3p region in NPC, resulting in the inactivation of many NPC-related tumor suppressor genes, which is closely related to the occurrence of NPC [12]. Our previous work has demonstrated that the overall survival rate and short-term efficacy of NPC patients with high expression of linc00312 were better than those with low expression of linc00312. Linc00312 can hinder the DNA damage signal sensation and transduction, inhibit the expression of DNA damage repair related proteins, and increase the radiosensitivity of NPC cells [13]. Overexpression of linc00312 was able to repress cell growth, inhibit invasion, and induce cell apoptosis in various cancers, including nonsmall cell lung cancer, thyroid cancer, colorectal cancer, and hepatocellular carcinoma [14–17]. A study reported linc00312 enhanced the sensitivity of ovarian cancer cells to cisplatin by promoting cell apoptosis [18].

Considering the important role of linc00312 in NPC, the aim of this study was to explore the relationship between genetic variations of linc00312 and the risk of chemoradiotherapy induced adverse reactions in NPC patients. This is of great importance to alleviate the toxic reactions of NPC patients by tailoring the treatment regimen according to individual genetic background.

## 2. Materials and Methods

### 2.1. Study Subjects

The NPC patients were recruited from the Hunan Provincial Cancer Hospital between October 2014 and July 2016. The inclusion criteria were as follows: (1) patients with pathologically confirmed NPC, (2) Han Chinese ethnicity, and (3) treatment with induction chemotherapy (IC) with platinum-based concurrent chemoradiotherapy (CCRT). Exclusion criteria were (1) underwent radiotherapy, chemotherapy, or surgery before; (2) the presence of distant metastasis or recurrence; and (3) had a second malignancy or with major organ dysfunction. This study was approved by the Ethics Committee of Central South University (CTXY-140007-2), and all patients signed informed consent before enrollment.

### 2.2. Treatment Regimen and Follow-Up

The patients were treated with platinum-based concurrent chemoradiotherapy (CCRT) plus induction chemotherapy (IC) scheme. The time span of treatment is 56 days~94 days. Radiation was administered five times per week with an accumulated radiation dose of 68-72 Gy to the primary tumor. Six chemotherapy regimens were included in this study: platinum plus 5-fluorouracil (FP), platinum plus paclitaxel (TP), platinum plus docetaxel (DP), platinum plus gemcitabine (GP), cisplatin alone (DDP), and nedaplatin alone (NDP). Every patient was evaluated for chemoradiotherapy induced toxic reactions weekly during treatment according to the Common Terminology Criteria for Adverse Events (CTCAE 3.0).

### 2.3. Candidate SNP Selection

ENCODE (http://genome.ucsc.edu/ENCODE/), ENSEMBL (http://asia.ensembl.org/index.html?redirect=no), and lncRNASNP (http://bioinfo.life.hust.edu.cn/lncRNASNP/) database were utilized to select SNPs with regulatory feature. We chose three SNPs of linc00312 (rs12497104, rs15734, and rs164966) that have not been reported before with a minor allele frequency (MAF) > 5% in the Chinese population for further study, as shown in Table 1.

### 2.4. DNA Extraction and Genotyping

3 ml of peripheral venous blood was collected from each patient before receiving any treatment. Genomic DNA was extracted using the QIAamp DNA Blood Mini Kit (Qiagen, Valencia, CA) according to the manufacturer's instructions. The concentration of DNA was measured using NanoDrop™ 1000 Spectrophotometer (Thermo Fisher Scientific, Wilmington, MA, USA). Genotyping was carried out by MassARRAY iPLEX platform (Sequenom, Inc., San Diego, CA, USA). The original data and genotyping map were obtained by using TYPER4.0 software. 10% of the samples were randomly set for repeated genotyping to assure the accuracy.

### 2.5. Statistical Analysis

SPSS 19.0 software (Chicago, IL, USA) was used for data statistical analysis. The Chi square test and Student's *t*-test were used to estimate the differences of variables between two groups. Univariate logistic and multivariate logistic regression were used to analysis the risk factors responsible for toxic reactions of NPC patients. The strength of the association between the risk factors and toxic reactions was evaluated by the ORs with 95% CIs. The genotype frequencies of the candidate SNPs were tested for deviation from the Hardy-Weinberg equilibrium (HWE). The SHEsis software was used for haplotype analysis. A *P* value of 0.05 or less was considered statistically significant.

## 3. Results

### 3.1. Population Characteristics

The demographic and clinical details of the 505 NPC patients included in this study were provided in Table S1, as we have previously reported [19]. Briefly, the average radiation dose was 71.34 Gy. DP (39.6%) and TP (40.2%) were the most commonly used IC regimens, and NDP (34.1%) and TP (21.4%) were the main CCRT regimens. According to CTCAE 3.0, 18.6% of patients experienced severe myelosuppression (grade > 2). Leucopenia (grade > 2, 43.0%) was the most frequent adverse event, followed by anemia (grade > 0, 19.4%), neutropenia (grade > 2, 14.5%), and thrombocytopenia (grade > 0, 10.1%). The genotyping information was shown in Table 1.

### 3.2. Association between Clinical Factors and Hematotoxicity in NPC Patients

We used univariate analysis to assess the influence of clinical factors, including gender, age, BMI, smoking, drinking, pathological type, clinical stage, IC regimen, CCRT regimen, and radiation dose, on the risk of chemoradiotherapy induced adverse reactions in NPC patients (Table 2). We revealed that high radiation dose (pGTVnx ≥ 71.00 Gy) was a risk factor for NPC patients suffering severe myelosuppression, severe neutropenia, and anemia with an OR of 1.657, 1.949, and 1.806, respectively. Women displayed 1.987 times and 3.097 times higher risk of occurring severe leukopenia and anemia during the treatment than men. Age was another risk factor for NPC patients as the elderly group (age > 47) showed 2.252 times and 2.299 times higher risk of anemia and thrombocytopenia than the younger group. Patients with advanced clinical stage were more prone to experience severe leucopenia with an OR of 4.437. Compared with smoking patients, the risk of anemia in nonsmoking patients decreased by 42.9%. In addition, chemotherapy regimens affect a variety of blood toxicity. However, none of the clinical factors presented any significant association with dermatitis and mucositis.

### 3.3. Linc00312 SNPs Were Associated with Hematotoxicity in NPC Patients

**Figure 1:**
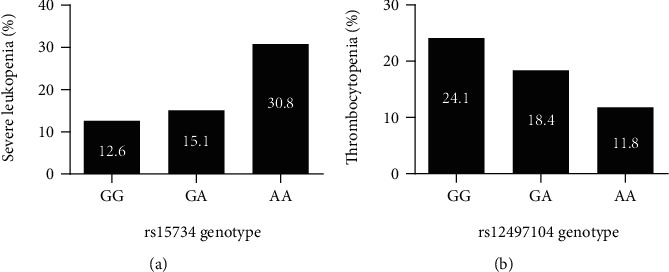
Gene-dose effect of selected SNPs and chemoradiotherapy induced toxicities. (a) Gene-dose effect of rs15734 on chemoradiotherapy induced severe leukopenia in NPC patients. (b) Gene-dose effect of rs1249714 on chemoradiotherapy induced thrombocytopenia in NPC patients.

Multivariate logistic regression adjusting clinical covariant was performed to identify the role of rs12497104, rs15734, and rs164966 on chemoradiotherapy induced adverse reactions. All of the three polymorphisms were in accordance with HWE and showed a call rate > 95%. Patients with rs15734 AA genotype displayed 3.145 times higher risk of severe leukopenia than GG genotype carriers (*P* = 0.029). Patients with rs12497104 AA genotype showed a 67.5% lower risk of thrombocytopenia than those with GG genotype (*P* = 0.030) (Table 3). Noteworthy, there exist a gene-does effect of rs15734 and rs12497104. As the copy number of rs15734 A risk allele increased, the incidence rate of severe leukopenia increased gradually from 12.6% to 15.1% to 30.8% for rs15734 GG vs. GA vs. AA genotype carriers. Similarly, with the increase of rs12497104 G risk allele, the incidence rate of thrombocytopenia increased from 11.8% to 18.4% to 24.1% for rs12497104 AA vs. GA vs. GG genotype carriers (Figure 1). Haplotype analysis of the three SNPs did not show any significant association with chemoradiotherapy induced toxic reactions (Table S2).

### 3.4. Prediction of Hematotoxicity Using Genetic and Clinical Information

We conducted an interaction analysis to assess the risk of toxic reactions before treatment by combining clinical factors (gender, age, and clinical stage) and genetic factors (rs15734 and rs12497104) (Tables 4 and 5). Compared to male with rs15734 GG genotype, the risk of severe leukopenia in male with rs15734 AA genotype increased to 3.876 times (*P* = 0.034), and the risk was remarkably increased to 5.635 times (*P* = 0.034) in female with rs15734 AA genotype. As we know, advanced stage (III-IV) was an unfavorable factor for severe leukopenia (OR = 10.120, *P* = 0.036). When we take rs15734 into consideration, the risk of severe leukopenia gradually increased from 10.120 to 11.385 to 25.241 following the addition of rs15734 A risk allele in patients with advanced stage. Older age (age ≥ 47) and rs12497104 G allele were risk factors for thrombocytopenia. Strikingly, the younger patients with rs12497104 AA genotype showed a 90% decreased risk of thrombocytopenia compared with older patients carrying rs12497104 GG genotype (*P* = 0.030).

### 3.5. Gene Expression Affected by Candidate SNPs

**Figure 2:**
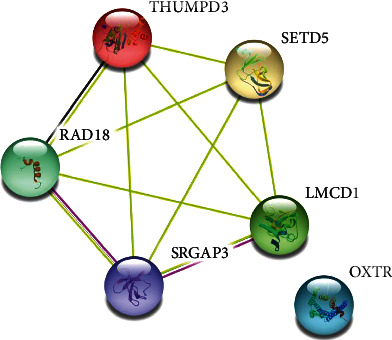
PPI network of genes regulated by rs12497104 and rs15734. The most significantly associated genes were LMCD1, OXTR, RAD18, THUMPD3, SRGAP3, and SETD5. Data was downloaded from Ensembl, and String database was utilized to construct a PPI network.

We tried to explore the rationale behind the association between the genotype and adverse reaction. Since rs12497104 and rs15734 were located in regulatory region with eQTL feature, we speculated that it might regulate gene expression in cis-acting or trans-acting pattern. We used Ensembl to analysis the gene expression affected by candidate SNPs. 886 gene expression correlations records were found with rs12497104 and rs15734 in different human tissues (Table S3-4). Among them, 91 gene expression correlation records were associated with blood cells. The most significantly associated genes were LMCD1, OXTR, RAD18, THUMPD3, SRGAP3, and SETD5. These genes were mainly enriched in PDGF signaling pathway (P00047). String database was utilized to construct a PPI network of these genes (Figure 2). The functions and implications of these genes will be discussed further.

## 4. Discussion

Hematotoxicity was the most frequently observed acute toxic reactions in NPC patients underwent chemoradiotherapy [20]. Over the past decades, clinicians and scientists in continued efforts to explore which regimen should be recommended for NPC patients with better efficacy and lesser toxicity. For example, Zhang et al. demonstrated that the IC + CCRT group had a higher incidence than the CCRT group of grade 3/4 neutropenia (28.0% vs. 10.5%), thrombocytopenia (11.3% vs. 1.3%), and anemia (9.6% vs. 0.8%) [1]. Tao et al. reported that the incidence rates of grade 3/4 leukocytopenia and neutropenia were significantly increased in the DP + DP group compared with the DP + DDP group in NPC patients [21].

It is crucial to work toward personalized precision treatment with more accurate risk stratification. In addition to anatomic TNM staging and parameters available in the clinic, exploration of additional novel factors, including comprehensive genomic profiling and other biomarkers, is urgently needed. Actually, a number of risk factors for treatment-related toxicity have been identified. Yu et al. reported that genetic polymorphisms of Wnt/*β*-catenin pathway, like GSK3*β* rs375557 and APC rs454886, were correlated with acute grade 3/4 dermatitis and oral mucositis of NPC patients [22]. Guo et al. demonstrated that genetic polymorphisms of NF-*κ*B pathway, such as CDKN2A rs3088440, CCND1 rs9344, and IKBKB rs12676482, were prone to suffering grade 3/4 acute treatment-induced myelosuppression of NPC patients [23]. A recent genome-wide association study revealed TNKS rs117157809 was associated with increased oral mucositis of NPC patients [24]. Our previous data indicated MYC rs4645948 was conferred with increased risk of anemia and severe leukopenia for NPC patients receiving chemoradiotherapy [25]. To date, most of the known toxic-related SNPs were located in protein-coding genes.

In the past decades, lncRNAs have attracted much attention since lncRNAs have been identified as vital regulator in multiple cellular biological processes. Genetic variants in lncRNAs have the potential to exert broad impact as they can affect their biogenesis, processing, and target site binding in a variety of ways [26]. Evidence have shown that lncRNA polymorphisms not only played a role in cancer susceptibility but also participate in regulating drug response. For example, SNP of lncRNA MIR2052HS affected the risk of breast cancer recurrence in women treated with aromatase inhibitors [27]. Previous study indicated that NPC patients carrying lncRNA GAS5 rs2067079 CT genotypes were more likely to experience chemoradiotherapy induced severe myelosuppression and severe neutropenia [19]. lncRNA MEG3 rs10132552 CC genotype carriers were related with increased risk of severe anemia when receiving radiochemotherapy [28].

In the present study, we found linc00312 rs15734 and rs12497104 were associated with severe leukopenia and thrombocytopenia in NPC patients during the treatment for the first time. Moreover, when we combine the genetic variations and clinical factors together to assess the risk of toxic reactions, the effect was even more remarkable. Our data indicated a 90% decreased risk of thrombocytopenia in the younger patients (age < 47) with rs12497104 AA genotype in contrast to older patients carrying rs12497104 GG genotype. Female with rs15734 AA genotype was exposed to 5.635-fold higher risk of experiencing severe leukopenia than male with rs15734 GG genotype. SNP is one of the most frequently observed type of genetic variants within lncRNA genes. The SNPs may play a role in certain disease by affecting the expression, secondary structure or function of lncRNAs. Our previous study has indicated that rs12497104 and rs15734 might destroy or create the binding site between a number of miRNAs and linc00312. Besides, it could affect the folding structure, minimum free energy, and expression level of linc00312 [29]. Considering the tumor suppressor role of linc00312 in NPC, we speculate that rs12497104 and rs15734 may affect the function of linc00312, thus participate in chemoradiotherapy induced hematotoxicity. However, no evidence has studied the function of linc00312 in normal blood cells or hematologic malignancies to date.

So, we conducted in silico analysis to explain the relationship between linc00312 gene variation and hematotoxicity. We found rs12497104 and rs15734 might regulate the expression of a number of genes in blood cells, for example, LMCD1, OXTR, RAD18, THUMPD3, SRGAP3, and SETD5. LMCD1 acts as transcriptional cofactor of GATA and functions in both hematopoietic and cardiovascular tissues [30]. LMCD1 could regulate differentiation of human bone marrow stem cells [31]. RAD18 is recruited to ionizing radiation- (IR-) induced DNA double-strand break (DSB) and proposed to propagate DNA DSB signaling and recruit DNA repair proteins [32]. Mutation of RAD18 results in defective replication of damaged DNA and hypersensitivity to multiple mutagens. It has been reported that polymorphisms of RAD18 were significantly associated with hematological toxicity in nonsmall cell lung cancer (NSCLC) [33]. THUMPD3 is a m2G methyltransferase that is involved in a broad range of tRNA methylation. THUMPD3 knockout cells exhibited impaired global protein synthesis and reduced growth [34]. SRGAP3 enables GTPase activator activity and participates in negative regulation of cell migration [35]. SETD5 may function as a histone methyltransferase and promote cell stemness of esophageal squamous cell carcinoma and NSCLC [36, 37]. OXTR belongs to the G-protein coupled receptor family and acts as a receptor for oxytocin. OXTR is known for its role in reproduction, social, and emotional behaviors [38]. We also conducted GO enrichment analysis of the regulated genes and revealed they were mainly involved in PDGF signaling pathway (P00047). PDGF signaling pathway is important for neutrophils, monocytes, and normal blood vessel formation. PDGF is shown to be chemotactic for monocytes and neutrophils and involved in inflammation [39, 40]. As we have mentioned above, we speculate rs12497104 and rs15734 may exert their function by regulating the expression of genes involved in hematological system. The underlying mechanism of the variations and radiochemotherapy induced toxicity still needs further *in vivo* and *in vitro* experimental studies to prove.

This is exactly exciting as we can predict the toxic reactions before treatment and tailor the regimen for individuals to reduce the adverse effect. Actually, Tang et al. have developed a gene expression-based signature to predict distant metastasis in locoregionally advanced NPC [41]. However, there was no prediction model for treatment-induced toxic reactions for NPC patients. Further studies with larger cohort in multicenter should be conducted to verify the present findings. Then, we can construct well-calibrated, integrated predictive models by combining genotyping profiles, clinical data, and treatment parameters, which may be helpful for each patient in selecting the best therapeutic approach and avoiding unnecessary worsening of quality of life.

In conclusion, we identified the association between genetic polymorphism of linc00312 and chemoradiotherapy induced hematotoxicity of NPC patients, which may provide some insight for personalized medicine.

## Figures and Tables

**Table 1:** Characteristics of the candidate SNPs.

SNP	Position	Regulatory feature	eQTL feature	MAF (CHS)	Call rate
rs12497104 (*G* > *A*)	Chr3:8573521	Open chromatin region	√	0.414	95.17%
rs15734 (*G* > *A*)	Chr3:8573803	Open chromatin region	√	0.229	99.75%
rs164966 (*A* > *G*)	Chr3:8573591	Promoter/enhancer	√	0.243	99.49%

**Table 2:** Univariate analysis of clinical characteristics and hematotoxicities in NPC patients receiving chemoradiotherapy.

Toxic reactions	Factors	OR (95% CI)	*P*
Myelosuppression	pGTVnx		
	<71.00 Gy	1 (reference)	
	≥71.00 Gy	1.657 (1.096-2.504)	0.017
	IC regimen		
	DP	1 (reference)	
	FP	0.986 (0.506-1.922)	0.967
	TP	2.549 (1.587-4.093)	<0.001
	GP	5.061 (1.387-18.469)	0.014
	CCRT regimen		
	FP	1 (reference)	
	TP	4.057 (2.040-8.068)	<0.001
	DDP	1.119 (0.502-2.491)	0.784
	NDP	0.986 (0.489-1.989)	0.969
	DP	1.979 (0.877-4.466)	0.100

Leukopenia	Gender		
	Male	1 (reference)	
	Female	1.987 (1.180-3.347)	0.010
	Clinical stage		
	I-II	1 (reference)	
	III-IV	4.437 (1.055-18.673)	0.042
	IC regimen		
	DP	1 (reference)	
	FP	1.710 (0.816-3.582)	0.155
	TP	2.054 (1.134-3.720)	0.018
	GP	6.351 (1.645-24.512)	0.007

Neutropenia	pGTVnx		
	<71.00 Gy	1 (reference)	
	≥71.00 Gy	1.949 (1.232-3.084)	0.004
	IC regimen		
	DP	1 (reference)	
	FP	0.551 (0.230-1.321)	0.182
	TP	2.613 (1.564-4.366)	<0.001
	GP	4.462 (1.179-16.879)	0.028
	CCRT regimen		
	FP	1 (reference)	
	TP	5.662 (2.478-12.935)	<0.001
	DDP	1.787 (0.699-4.568)	0.225
	NDP	1.266 (0.533-3.006)	0.592
	DP	2.844 (1.094-7.393)	0.032

Anemia	Gender		
	Male	1 (reference)	
	Female	3.097 (2.048-4.683)	<0.001
	Age		
	<47	1 (reference)	
	≥47	2.252 (1.565-3.241)	<0.001
	Smoking status		
	Smoker	1 (reference)	
	Nonsmoker	0.572 (0.401-0.817)	0.002
	pGTVnx		
	<71.00 Gy	1 (reference)	
	≥71.00 Gy	1.806 (1.265-2.579)	0.001
	IC regimen		
	DP	1 (reference)	
	FP	1.095 (0.659-1.819)	0.727
	TP	1.653 (1.111-2.460)	0.013
	GP	3.973 (0.997-15.834)	0.051
	CCRT regimen		
	FP	1 (reference)	
	TP	2.232 (1.248-3.993)	0.007
	DDP	1.780 (0.963-3.292)	0.066
	NDP	0.718 (0.416-1.239)	0.234
	DP	1.599 (0.810-3.156)	0.176

Thrombocytopenia	Age		
	<47	1 (reference)	
	≥47	2.299 (1.429-3.698)	0.001
	IC regimen		
	DP	1 (reference)	
	FP	1.571 (0.866-2.853)	0.137
	TP	0.982 (0.587-1.644)	0.945
	GP	4.714 (1.295-17.163)	0.019

**Table 3:** Multivariate logistic analysis of candidate SNPs and concurrent chemoradiotherapy induced hematotoxicities in NPC patients.

Genotypes	Leukopenia		OR (95% CI)	*P*	Thrombocytopenia		OR (95% CI)	*P*
	Grade ≤ 2 *N* (%)	Grade > 2 *N* (%)			Grade 0 *N* (%)	Grade > 0 *N* (%)		
rs15734 (*G* > *A*)								
GG	256 (59.3)	37 (50.7)	1.00 (reference)		240 (59.0)	53 (54.1)	1.00 (reference)	
GA	157 (36.3)	28 (38.4)	1.155 (0.648-2.058)	0.626	146 (35.9)	39 (39.8)	1.295 (0.791-2.122)	0.304
AA	18 (4.2)	8 (11.0)	3.145 (1.123-8.806)	0.029	20 (4.9)	6 (6.1)	1.293 (0.473-3.530)	0.617
AA + GA vs. GG			1.353 (0.787-2.327)	0.274			1.295 (0.808-2.075)	0.283
AA vs. GA + GG			2.986 (1.090-8.178)	0.033			1.175 (0.439-3.150)	0.748
rs12497104 (*G* > *A*)								
GG	145 (33.6)	29 (39.7)	1.00 (reference)		132 (32.4)	42 (42.9)	1.00 (reference)	
GA	199 (46.1)	29 (39.7)	0.725 (0.397-1.324)	0.296	186 (45.7)	42 (42.9)	0.686 (0.411-1.146)	0.150
AA	42 (9.7)	9 (12.3)	0.836 (0.334-2.097)	0.703	45 (11.1)	6 (6.1)	0.325 (0.118-0.895)	0.030
AA + GA vs. GG			0.747 (0.422-1.321)	0.316			0.613 (0.373-1.009)	0.054
AA vs. GA + GG			0.993 (0.418-2.357)	0.987			0.399 (0.150-1.060)	0.065
rs164966 (*A* > *G*)								
AA	249 (57.6)	37 (50.7)	1.00 (reference)		235 (57.7)	51 (52.0)	1.00 (reference)	
GA	156 (36.1)	28 (38.4)	1.088 (0.609-1.941)	0.776	146 (35.9)	38 (38.8)	1.229 (0.746-2.025)	0.419
GG	23 (5.3)	8 (11.0)	2.554 (0.941-6.930)	0.066	24 (5.9)	7 (7.1)	1.228 (0.483-3.125)	0.666
GG + GA vs. AA			1.257 (0.731-2.161)	0.408			1.229 (0.765-1.974)	0.395
GG vs. GA + AA			2.293 (0.885-5.944)	0.088			1.135 (0.456-2.827)	0.785

**Table 4:** Prediction of the risk of leukopenia in NPC patients based on the interaction between rs15734, gender, and clinical stages.

Clinical factors	Genotypes	Leukopenia		OR (95% CI)	*P*
		Grade ≤ 2 *N* (%)	Grade > 2 *N* (%)		
Gender	rs15734 (*G* > *A*)				
Male	GG	189 (90.4)	20 (9.6)	1.00 (reference)	
Male	GA	126 (86.3)	20 (13.7)	1.385 (0.694-2.764)	0.356
Male	AA	14 (73.7)	5 (26.3)	3.876 (1.108-13.563)	0.034
Female	GG	67 (79.8)	17 (20.2)	2.992 (1.412-6.339)	0.004
Female	GA	31 (79.5)	8 (20.5)	2.068 (0.794-5.384)	0.137
Female	AA	5 (62.5)	3 (37.5)	5.635 (1.140-27.857)	0.034
Clinical stage					
I-II	GG	30 (96.8)	1 (3.2)	1.00 (reference)	
I-II	GA	16 (100.0)	0 (0.0)	—	**—**
I-II	AA	2 (66.7)	1 (33.3)	18.481 (0.585-584.166)	0.098
III-IV	GG	226 (86.3)	36 (13.7)	10.120 (1.159-88.373)	0.036
III-IV	GA	141 (83.4)	28 (16.6)	11.385 (1.299-99.802)	0.028
III-IV	AA	17 (70.8)	7 (29.2)	25.241 (2.368-269.073)	0.007

**Table 5:** Prediction of the risk of thrombocytopenia in NPC patients based on the interaction between rs12497104 and age.

Clinical factors	Genotypes	Thrombocytopenia		OR (95% CI)	*P*
		Grade 0 *N* (%)	Grade > 0 *N* (%)		
Age	rs12497104 (*G* > *A*)				
≥47	GG	70 (68.0)	33 (32.0)	1.00 (reference)	
≥47	GA	95 (79.2)	25 (20.8)	0.520 (0.278-0.971)	0.040
≥47	AA	28 (84.8)	5 (15.2)	0.344 (0.114-1.038)	0.058
<47	GG	62 (87.3)	9 (12.7)	0.276 (0.120-0.635)	0.002
<47	GA	91 (84.3)	17 (15.7)	0.381 (0.192-0.755)	0.006
<47	AA	17 (94.4)	1 (5.6)	0.105 (0.013-0.883)	0.038

## Data Availability

The data used to support the findings of this study are included within the article and supplementary file.

## Supplementary Materials

Supplementary 1 Supplementary Table 1: demographic and clinical details of the NPC patients.

Supplementary 2 Supplementary Table 2: haplotype analysis of the selected polymorphisms.

Supplementary 3 Supplementary Table 3: data from Ensembl indicates the gene expression correlated with rs12497104.

Supplementary 4 Supplementary Table 4: data from Ensembl indicates the gene expression correlated with rs15734.
