# Supplementary material for: Linc00312 Single Nucleotide Polymorphism as Biomarker for Chemoradiotherapy Induced Hematotoxicity in Nasopharyngeal Carcinoma Patients
Source: Dis Markers. 2022 Aug 8;2022:6707821. doi: 10.1155/2022/6707821 (PMC9381851; doi:10.1155/2022/6707821)
Supplement: Supplementary 1 — Supplementary Table 1: demographic and clinical details of the NPC patients. [file 6707821.f1.docx]

**Supplementary Table 1. Demographic and clinical details of the NPC patients.**

| Patient characteristics | N=505 (%) | Patient characteristics | N=505 (%) |
| --- | --- | --- | --- |
| Gender |  | N-staging |  |
| Male | 374 (74.1) | N0-N1 | 93 (18.4) |
| Female | 131 (25.9) | N2-N3 | 412 (81.6) |
| Age, years |  | IC regimen |  |
| Mean±SD | 47.41±9.15 | DP | 200 (39.6) |
| < 47 | 229 (45.3) | FP | 92 (18.2) |
| ≥ 47 | 276 (54.7) | TP | 203 (40.2) |
| BMI |  | GP | 10 (2) |
| < 18.5 | 30 (5.9) | CCRT regimen |  |
| 18.5 ~ 24 | 274 (54.3) | FP | 85 (16.8) |
| ≥ 24 | 201 (39.8) | TP | 108 (21.4) |
| Smoking status |  | DDP | 83 (16.4) |
| Smoker | 247 (48.9) | NDP | 172 (34.1) |
| Nonsmoker | 258 (51.1) | DP | 57 (11.3) |
| Drinking status |  | pGTVnx (irradiation dose) |  |
| Drinker | 90 (17.8) | Mean±SD | 71.34±2.79 |
| Nondrinker | 415 (82.2) | < 71.00Gy | 261 (51.7) |
| Histological type |  | ≥71.00Gy | 234 (48.3) |
| WHO type II | 214 (42.4) | Toxic reactions | 51 (10.1) |
| WHO type III | 291 (57.6) | Grade 3-4 Myelosuppression | 94 (18.6) |
| Clinical stage |  | Grade 3-4 Neutropenia | 73 (14.5) |
| I-II | 50 (9.9) | Grade 3-4 Leukopenia | 217 (43.0) |
| III-IV | 455 (90.1) | Anemia | 98 (19.4) |
| T-staging |  | Thrombocytopenia | 51 (10.1) |
| T1-T2 | 246 (48.7) |  |  |
| T3-T4 | 259 (51.3) |  |  |

Abbreviations: BMI, Body mass index; CCRT, Concurrent chemoradiotherapy; IC, Induction chemotherapy; DP, Docetaxel + Cisplatin/Nedaplatin; FP, 5-fluorouracil + Cisplatin/Nedaplatin; TP, Paclitaxel + Cisplatin/Nedaplatin; GP, Gemcitabine + Cisplatin/Nedaplatin; DDP, Cisplatin alone; NDP, Nedaplatin alone.
