# Supplementary material for: Linc00312 Single Nucleotide Polymorphism as Biomarker for Chemoradiotherapy Induced Hematotoxicity in Nasopharyngeal Carcinoma Patients
Source: Dis Markers. 2022 Aug 8;2022:6707821. doi: 10.1155/2022/6707821 (PMC9381851; doi:10.1155/2022/6707821)
Supplement: Supplementary 2 — Supplementary Table 2: haplotype analysis of the selected polymorphisms. [file 6707821.f2.docx]

**Supplementary Table 2. Haplotype analysis of the selected polymorphisms.**

| **Toxic reactions** | **Haplotype**  **(rs12497104-rs164966-rs15734)** | **Control^#^**  **(freq)** | **Case^#^**  **(freq)** | **OR [95% CI]** | ***P*** |
| --- | --- | --- | --- | --- | --- |
| **Myelosuppression** | A A G | 249.98(0.372) | 77.41(0.346) | 0.886 [0.645~1.217] | 0.456 |
|  | A G A | 0.02(0.000) | 1.59(0.007) | - | - |
|  | G A G | 262.02(0.390) | 79.59(0.355) | 0.857 [0.625~1.174] | 0.337 |
|  | G G A | 152.97(0.228) | 65.41(0.292) | 1.394 [0.992~1.960] | 0.055 |
|  | G G G | 7.00(0.010) | 0.00(0.000) | - | - |
|  |  |  |  |  |  |
| **Leukopenia** | A A G | 281.97(0.370) | 45.48(0.339) | 0.877 [0.595~1.292] | 0.507 |
|  | A G A | 0.03(0.000) | 1.52(0.011) | - | **-** |
|  | G A G | 295.03(0.387) | 46.52(0.347) | 0.844 [0.574~1.241] | 0.387 |
|  | G G A | 177.97(0.234) | 40.48(0.302) | 1.427 [0.950~2.141] | 0.085 |
|  | G G G | 7.00(0.009) | 0.00(0.000) | - | **-** |
|  |  |  |  |  |  |
| **Neutropenia** | A A G | 264.17(0.366) | 63.00(0.362) | 0.965 [0.684~1.361] | 0.838 |
|  | A G A | 1.83(0.003) | 0.00(0.000) | - | **-** |
|  | G A G | 282.83(0.392) | 59.00(0.339) | 0.781 [0.551~1.105] | 0.162 |
|  | G G A | 166.17(0.230) | 52.00(0.299) | 1.403 [0.971~2.027] | 0.070 |
|  | G G G | 7.00(0.010) | 0.00(0.000) | - | **-** |
|  |  |  |  |  |  |
| **Anemia** | A A G | 170.19(0.339) | 156.99(0.398) | 1.285 [0.977~1.691] | 0.073 |
|  | A G A | 1.81(0.004) | 0.01(0.000) | - | **-** |
|  | G A G | 196.81(0.392) | 145.01(0.368) | 0.897 [0.683~1.178] | 0.434 |
|  | G G A | 129.19(0.257) | 88.99(0.226) | 0.837 [0.614~1.141] | 0.261 |
|  | G G G | 4.00(0.008) | 3.00(0.008) | - | **-** |
|  |  |  |  |  |  |
| **Thrombocytopenia** | A A G | 273.30(0.380) | 53.99(0.307) | 0.717 [0.503~1.022] | 0.065 |
|  | A G A | 1.70(0.002) | 0.01(0.000) | - | - |
|  | G A G | 270.70(0.376) | 71.01(0.403) | 1.114 [0.795~1.561] | 0.531 |
|  | G G A | 168.30(0.234) | 49.99(0.284) | 1.293 [0.892~1.873] | 0.174 |
|  | G G G | 6.00(0.008) | 1.00(0.006) | - | - |

Note: All those frequency<0.03 will be ignored in analysis.

# Case refers to myelosuppression (Grade>2), neutropenia (Grade>2), leukopenia (Grade>2), anemia (Grade>0) and thrombocytopenia (Grade>0). Control refers to myelosuppression (Grade≤2), neutropenia (Grade≤2), leukopenia (Grade≤2), anemia (Grade=0) and thrombocytopenia (Grade=0).
