# Supplementary material for: Linc00312 Single Nucleotide Polymorphism as Biomarker for Chemoradiotherapy Induced Hematotoxicity in Nasopharyngeal Carcinoma Patients
Source: Dis Markers. 2022 Aug 8;2022:6707821. doi: 10.1155/2022/6707821 (PMC9381851; doi:10.1155/2022/6707821)
Supplement: Supplementary 3 — Supplementary Table 3: data from Ensembl indicates the gene expression correlated with rs12497104. [file 6707821.f3.docx]

| **Supplementary Table 3. Data from Ensembl indicates the gene expression correlated with rs12497104.** | | | |
| --- | --- | --- | --- |
| **Gene** | **P-value (-log10)** | **Effect size** | **Tissue** |
| ENSG00000070950 | 0.793889545 | -0.0472532 | macrophage_IFNg |
| ENSG00000071282 | 0.228223705 | 0.070134 | macrophage_IFNg |
| ENSG00000134077 | 0.102886119 | -0.038102 | macrophage_IFNg |
| ENSG00000168137 | 0.033023816 | 0.00193347 | macrophage_IFNg |
| ENSG00000180914 | 0.477609241 | -0.16407 | macrophage_IFNg |
| ENSG00000206573 | 0.361658528 | 0.0419248 | macrophage_IFNg |
| ENSG00000125046 | 0.194893516 | 0.17219 | macrophage_IFNg |
| ENSG00000070950 | 0.384394997 | 0.0253492 | macrophage_naive |
| ENSG00000071282 | 0.282579996 | 0.0688764 | macrophage_naive |
| ENSG00000134077 | 0.037463988 | -0.0150825 | macrophage_naive |
| ENSG00000168137 | 1.07487002 | -0.0299532 | macrophage_naive |
| ENSG00000180914 | 0.231310112 | -0.089242 | macrophage_naive |
| ENSG00000196220 | 0.68959348 | -0.179292 | macrophage_naive |
| ENSG00000206573 | 0.189996791 | 0.0243205 | macrophage_naive |
| ENSG00000070950 | 0.192559688 | 0.0174693 | macrophage_Salmonella |
| ENSG00000071282 | 0.000211987 | 8.06E-05 | macrophage_Salmonella |
| ENSG00000134077 | 0.028382789 | -0.0109216 | macrophage_Salmonella |
| ENSG00000168137 | 0.686434361 | 0.0273795 | macrophage_Salmonella |
| ENSG00000180914 | 0.740040779 | -0.153137 | macrophage_Salmonella |
| ENSG00000206573 | 0.808286364 | 0.0757866 | macrophage_Salmonella |
| ENSG00000070950 | 0.381401797 | 0.0172668 | monocyte |
| ENSG00000134077 | 0.75076739 | -0.0345401 | monocyte |
| ENSG00000168137 | 0.111600886 | -0.0037052 | monocyte |
| ENSG00000196220 | 0.339350775 | -0.0320029 | monocyte |
| ENSG00000206573 | 0.714976323 | -0.0469352 | monocyte |
| ENSG00000270207 | 0.132826659 | 0.0158092 | monocyte |
| ENSG00000070950 | 0.267864481 | 0.0126083 | neutrophil |
| ENSG00000134077 | 0.15055233 | 0.0130585 | neutrophil |
| ENSG00000168137 | 0.338956289 | 0.00881597 | neutrophil |
| ENSG00000206573 | 0.074322883 | -0.00382405 | neutrophil |
| ENSG00000070950 | 0.023250764 | 0.000831239 | T-cell |
| ENSG00000134077 | 0.705632989 | -0.0167657 | T-cell |
| ENSG00000168137 | 0.047276798 | 0.00117748 | T-cell |
| ENSG00000196220 | 0.126549188 | 0.0122088 | T-cell |
| ENSG00000206573 | 0.908860846 | -0.0419435 | T-cell |
| ENSG00000254485 | 0.004503012 | -0.0010513 | T-cell |
| ENSG00000070950 | 0.085845201 | -0.00471087 | brain |
| ENSG00000071282 | 0.130266784 | 0.00943706 | brain |
| ENSG00000134077 | 0.419485575 | -0.0099104 | brain |
| ENSG00000156959 | 0.380920241 | 0.015365 | brain |
| ENSG00000168137 | 0.218853782 | -0.00684478 | brain |
| ENSG00000180914 | 0.002206601 | 0.000321448 | brain |
| ENSG00000196220 | 0.004855967 | -0.000187068 | brain |
| ENSG00000206573 | 1.067647002 | -0.0239236 | brain |
| ENSG00000224808 | 0.162640131 | -0.0136053 | brain |
| ENSG00000224884 | 0.036101725 | -0.00388904 | brain |
| ENSG00000227110 | 0.560981691 | -0.032368 | brain |
| ENSG00000235830 | 0.036750654 | -0.00403006 | brain |
| ENSG00000254485 | 0.86111968 | -0.0404326 | brain |
| ENSG00000070950 | 0.010768719 | 0.000481218 | adipose_naive |
| ENSG00000071282 | 0.536074179 | -0.0284994 | adipose_naive |
| ENSG00000125046 | 0.580969971 | -0.0695999 | adipose_naive |
| ENSG00000134077 | 0.014900605 | 0.000536207 | adipose_naive |
| ENSG00000168137 | 0.0159919 | -0.000539344 | adipose_naive |
| ENSG00000182533 | 1.269588405 | 0.201748 | adipose_naive |
| ENSG00000196220 | 0.521946014 | 0.030672 | adipose_naive |
| ENSG00000206573 | 0.665680981 | 0.0220274 | adipose_naive |
| ENSG00000070950 | 0.139932838 | 0.00554411 | muscle_naive |
| ENSG00000071282 | 0.017033999 | -0.00133298 | muscle_naive |
| ENSG00000134077 | 0.502571338 | -0.00889069 | muscle_naive |
| ENSG00000168137 | 0.095296353 | 0.00248283 | muscle_naive |
| ENSG00000182533 | 0.806675004 | -0.0254059 | muscle_naive |
| ENSG00000196220 | 0.013129777 | -0.00108552 | muscle_naive |
| ENSG00000206573 | 1.156320073 | 0.0257002 | muscle_naive |
| ENSG00000224884 | 0.049951731 | -0.0127762 | muscle_naive |
| ENSG00000235830 | 0.242323137 | -0.0592887 | muscle_naive |
| ENSG00000070950 | 0.160250525 | -0.0115528 | fibroblast |
| ENSG00000071282 | 0.529107554 | -0.0733445 | fibroblast |
| ENSG00000134077 | 0.502474643 | -0.0175118 | fibroblast |
| ENSG00000168137 | 0.787828382 | 0.0242763 | fibroblast |
| ENSG00000180914 | 0.411676741 | -0.0963774 | fibroblast |
| ENSG00000206573 | 0.240003726 | -0.0210928 | fibroblast |
| ENSG00000070950 | 0.612152981 | 0.0215524 | LCL |
| ENSG00000071282 | 0.385784275 | -0.10476 | LCL |
| ENSG00000134077 | 0.31808161 | -0.0106831 | LCL |
| ENSG00000168137 | 0.476178123 | -0.0129502 | LCL |
| ENSG00000180914 | 0.826609749 | 0.0819284 | LCL |
| ENSG00000206573 | 1.136256138 | -0.0488638 | LCL |
| ENSG00000254485 | 0.433582544 | -0.088064 | LCL |
| ENSG00000071282 | 10.38122311 | -0.652942 | T-cell |
| ENSG00000180914 | 0.524706623 | 0.0575392 | T-cell |
| ENSG00000224808 | 0.155790691 | 0.0239359 | T-cell |
| ENSG00000227929 | 0.918123651 | 0.108971 | T-cell |
| ENSG00000228723 | 0.093146378 | 0.0184984 | T-cell |
| ENSG00000235830 | 0.191903188 | -0.0230318 | T-cell |
| ENSG00000196220 | 1.243325864 | -0.108649 | LCL |
| ENSG00000206573 | 0.209500205 | 0.0163408 | Adipose_Subcutaneous |
| ENSG00000180914 | 0.252379253 | 0.0335228 | Adipose_Subcutaneous |
| ENSG00000168137 | 0.103332164 | -0.00683615 | Adipose_Subcutaneous |
| ENSG00000227110 | 0.004356032 | 0.00065446 | Adipose_Subcutaneous |
| ENSG00000125046 | 0.126756729 | -0.0133281 | Adipose_Subcutaneous |
| ENSG00000070950 | 0.333126493 | 0.0297401 | Adipose_Subcutaneous |
| ENSG00000196220 | 0.092612827 | 0.0100146 | Adipose_Subcutaneous |
| ENSG00000071282 | 1.845685603 | 0.0819296 | Adipose_Subcutaneous |
| ENSG00000182533 | 0.852302039 | 0.0762276 | Adipose_Subcutaneous |
| ENSG00000134077 | 1.04693022 | 0.0571204 | Adipose_Subcutaneous |
| ENSG00000206573 | 1.983798463 | 0.100759 | Adipose_Visceral_Omentum |
| ENSG00000156959 | 0.069856152 | -0.0104356 | Adipose_Visceral_Omentum |
| ENSG00000070950 | 0.640094353 | 0.0511453 | Adipose_Visceral_Omentum |
| ENSG00000180914 | 0.486064942 | 0.0588406 | Adipose_Visceral_Omentum |
| ENSG00000182533 | 0.081448089 | 0.0102204 | Adipose_Visceral_Omentum |
| ENSG00000071282 | 1.053993558 | 0.0520505 | Adipose_Visceral_Omentum |
| ENSG00000134077 | 0.652217383 | -0.0437796 | Adipose_Visceral_Omentum |
| ENSG00000227110 | 0.320332308 | 0.0377164 | Adipose_Visceral_Omentum |
| ENSG00000196220 | 0.227370288 | 0.0241518 | Adipose_Visceral_Omentum |
| ENSG00000168137 | 0.053940448 | -0.00406464 | Adipose_Visceral_Omentum |
| ENSG00000125046 | 0.087128317 | -0.00973617 | Adipose_Visceral_Omentum |
| ENSG00000235830 | 0.126277266 | 0.0300034 | Adrenal_Gland |
| ENSG00000134077 | 0.188114129 | -0.023043 | Adrenal_Gland |
| ENSG00000254485 | 2.395459683 | -0.294937 | Adrenal_Gland |
| ENSG00000071282 | 0.320199751 | -0.0502168 | Adrenal_Gland |
| ENSG00000227110 | 0.005059794 | -0.00124541 | Adrenal_Gland |
| ENSG00000168137 | 0.681239848 | -0.0590957 | Adrenal_Gland |
| ENSG00000070950 | 0.068630116 | 0.0127716 | Adrenal_Gland |
| ENSG00000206573 | 0.943842473 | 0.0822234 | Adrenal_Gland |
| ENSG00000156959 | 0.871000114 | 0.0914619 | Adrenal_Gland |
| ENSG00000196220 | 0.011685458 | -0.00248294 | Adrenal_Gland |
| ENSG00000228723 | 0.207683268 | -0.0375199 | Adrenal_Gland |
| ENSG00000125046 | 0.416910097 | 0.0689759 | Adrenal_Gland |
| ENSG00000180914 | 0.496319606 | 0.0779833 | Adrenal_Gland |
| ENSG00000180914 | 1.000721093 | 0.0869233 | Artery_Aorta |
| ENSG00000156959 | 0.804745353 | 0.0985735 | Artery_Aorta |
| ENSG00000182533 | 0.317126895 | -0.0567887 | Artery_Aorta |
| ENSG00000206573 | 0.253949923 | -0.0151941 | Artery_Aorta |
| ENSG00000196220 | 0.16379302 | -0.0222923 | Artery_Aorta |
| ENSG00000125046 | 1.03340496 | -0.120502 | Artery_Aorta |
| ENSG00000227110 | 0.187109361 | 0.0234788 | Artery_Aorta |
| ENSG00000168137 | 0.079867278 | -0.00408715 | Artery_Aorta |
| ENSG00000134077 | 0.009968546 | -0.000957072 | Artery_Aorta |
| ENSG00000071282 | 0.246873688 | 0.0212344 | Artery_Aorta |
| ENSG00000070950 | 1.275392391 | -0.101477 | Artery_Aorta |
| ENSG00000227110 | 0.570439885 | 0.0887754 | Artery_Coronary |
| ENSG00000125046 | 0.299562824 | -0.0598349 | Artery_Coronary |
| ENSG00000182533 | 0.819326582 | -0.0973234 | Artery_Coronary |
| ENSG00000196220 | 0.291859655 | 0.0498991 | Artery_Coronary |
| ENSG00000134077 | 0.619993287 | 0.0597814 | Artery_Coronary |
| ENSG00000168137 | 0.07270716 | -0.00819598 | Artery_Coronary |
| ENSG00000206573 | 0.641872811 | -0.0703125 | Artery_Coronary |
| ENSG00000071282 | 0.818442226 | 0.1122 | Artery_Coronary |
| ENSG00000156959 | 0.347338908 | 0.0789689 | Artery_Coronary |
| ENSG00000070950 | 0.163263943 | -0.0319752 | Artery_Coronary |
| ENSG00000180914 | 0.179769632 | -0.0409472 | Artery_Coronary |
| ENSG00000070950 | 0.335075167 | 0.0286532 | Artery_Tibial |
| ENSG00000182533 | 0.020490322 | -0.00234612 | Artery_Tibial |
| ENSG00000134077 | 0.302158019 | -0.0198293 | Artery_Tibial |
| ENSG00000125046 | 0.569554049 | -0.0527205 | Artery_Tibial |
| ENSG00000156959 | 0.368008635 | 0.0426343 | Artery_Tibial |
| ENSG00000180914 | 0.118340358 | -0.0133622 | Artery_Tibial |
| ENSG00000196220 | 1.091823386 | -0.0835523 | Artery_Tibial |
| ENSG00000227110 | 0.739666206 | 0.0573978 | Artery_Tibial |
| ENSG00000168137 | 0.662235342 | 0.0203969 | Artery_Tibial |
| ENSG00000071282 | 0.17449534 | 0.0133366 | Artery_Tibial |
| ENSG00000206573 | 0.306809657 | 0.0159916 | Artery_Tibial |
| ENSG00000180914 | 0.093724772 | 0.0306882 | Brain_Amygdala |
| ENSG00000134077 | 0.503300008 | 0.0906963 | Brain_Amygdala |
| ENSG00000254485 | 0.090965758 | 0.0314287 | Brain_Amygdala |
| ENSG00000235830 | 0.575667293 | 0.138662 | Brain_Amygdala |
| ENSG00000070950 | 0.146166776 | 0.0434467 | Brain_Amygdala |
| ENSG00000196220 | 0.125167525 | 0.020273 | Brain_Amygdala |
| ENSG00000071282 | 0.557365042 | 0.134431 | Brain_Amygdala |
| ENSG00000227110 | 0.1340218 | 0.0303628 | Brain_Amygdala |
| ENSG00000270207 | 0.128161549 | -0.0405899 | Brain_Amygdala |
| ENSG00000125046 | 0.386461551 | 0.0901003 | Brain_Amygdala |
| ENSG00000206573 | 0.161389522 | 0.0403646 | Brain_Amygdala |
| ENSG00000156959 | 0.26792484 | -0.0275022 | Brain_Amygdala |
| ENSG00000168137 | 0.244342346 | -0.0364755 | Brain_Amygdala |
| ENSG00000206573 | 0.446234661 | -0.101608 | Brain_Anterior_cingulate_cortex_BA24 |
| ENSG00000214041 | 0.499029798 | -0.0913164 | Brain_Anterior_cingulate_cortex_BA24 |
| ENSG00000134077 | 0.536190595 | 0.09022 | Brain_Anterior_cingulate_cortex_BA24 |
| ENSG00000070950 | 0.22097236 | 0.0490151 | Brain_Anterior_cingulate_cortex_BA24 |
| ENSG00000196220 | 1.157908353 | 0.0806235 | Brain_Anterior_cingulate_cortex_BA24 |
| ENSG00000270207 | 0.6473352 | -0.14172 | Brain_Anterior_cingulate_cortex_BA24 |
| ENSG00000227110 | 1.443396011 | -0.156151 | Brain_Anterior_cingulate_cortex_BA24 |
| ENSG00000156959 | 0.055373738 | -0.00481272 | Brain_Anterior_cingulate_cortex_BA24 |
| ENSG00000254485 | 0.291627546 | 0.0827978 | Brain_Anterior_cingulate_cortex_BA24 |
| ENSG00000235830 | 0.025730622 | -0.00810828 | Brain_Anterior_cingulate_cortex_BA24 |
| ENSG00000071282 | 0.442089969 | 0.095238 | Brain_Anterior_cingulate_cortex_BA24 |
| ENSG00000182533 | 0.191012315 | 0.0557206 | Brain_Anterior_cingulate_cortex_BA24 |
| ENSG00000180914 | 0.173522203 | 0.0355606 | Brain_Anterior_cingulate_cortex_BA24 |
| ENSG00000125046 | 1.170422869 | -0.20253 | Brain_Anterior_cingulate_cortex_BA24 |
| ENSG00000168137 | 0.327809749 | -0.049542 | Brain_Anterior_cingulate_cortex_BA24 |
| ENSG00000156959 | 0.473711135 | -0.0251728 | Brain_Caudate_basal_ganglia |
| ENSG00000134077 | 0.500396761 | -0.0678548 | Brain_Caudate_basal_ganglia |
| ENSG00000168137 | 0.024651841 | -0.00396525 | Brain_Caudate_basal_ganglia |
| ENSG00000125046 | 0.127111 | -0.0280495 | Brain_Caudate_basal_ganglia |
| ENSG00000071282 | 0.249314221 | -0.039105 | Brain_Caudate_basal_ganglia |
| ENSG00000196220 | 0.316408183 | -0.0424143 | Brain_Caudate_basal_ganglia |
| ENSG00000206573 | 0.853868862 | -0.0925512 | Brain_Caudate_basal_ganglia |
| ENSG00000182533 | 0.364704287 | 0.0801988 | Brain_Caudate_basal_ganglia |
| ENSG00000070950 | 0.560845798 | 0.08988 | Brain_Caudate_basal_ganglia |
| ENSG00000270207 | 0.537356471 | 0.116652 | Brain_Caudate_basal_ganglia |
| ENSG00000235830 | 0.022298796 | 0.00637741 | Brain_Caudate_basal_ganglia |
| ENSG00000254485 | 0.04851774 | -0.0151689 | Brain_Caudate_basal_ganglia |
| ENSG00000180914 | 0.002865352 | 0.000714517 | Brain_Caudate_basal_ganglia |
| ENSG00000227110 | 0.368183993 | 0.0621125 | Brain_Caudate_basal_ganglia |
| ENSG00000070950 | 0.426231705 | 0.0666801 | Brain_Cerebellar_Hemisphere |
| ENSG00000071282 | 0.550479948 | 0.0609319 | Brain_Cerebellar_Hemisphere |
| ENSG00000196220 | 0.028254847 | 0.00460072 | Brain_Cerebellar_Hemisphere |
| ENSG00000125046 | 0.317650014 | 0.084478 | Brain_Cerebellar_Hemisphere |
| ENSG00000227929 | 0.393680989 | 0.0917607 | Brain_Cerebellar_Hemisphere |
| ENSG00000134077 | 0.702670716 | -0.0839523 | Brain_Cerebellar_Hemisphere |
| ENSG00000180914 | 0.941327287 | -0.123151 | Brain_Cerebellar_Hemisphere |
| ENSG00000227110 | 0.610127211 | -0.0936129 | Brain_Cerebellar_Hemisphere |
| ENSG00000228723 | 0.059798393 | -0.0177709 | Brain_Cerebellar_Hemisphere |
| ENSG00000182533 | 0.939596825 | -0.162822 | Brain_Cerebellar_Hemisphere |
| ENSG00000206573 | 0.238465308 | -0.0346731 | Brain_Cerebellar_Hemisphere |
| ENSG00000156959 | 0.484053339 | 0.060887 | Brain_Cerebellar_Hemisphere |
| ENSG00000168137 | 0.678776587 | -0.0603735 | Brain_Cerebellar_Hemisphere |
| ENSG00000254485 | 0.03872322 | -0.0137168 | Brain_Cerebellar_Hemisphere |
| ENSG00000270207 | 0.862816855 | 0.165879 | Brain_Cerebellar_Hemisphere |
| ENSG00000196220 | 0.240345751 | 0.0325249 | Brain_Cerebellum |
| ENSG00000227929 | 0.037532639 | -0.0111061 | Brain_Cerebellum |
| ENSG00000228723 | 1.432995996 | 0.2149 | Brain_Cerebellum |
| ENSG00000206573 | 0.088809887 | -0.0137455 | Brain_Cerebellum |
| ENSG00000134077 | 0.262196911 | -0.036665 | Brain_Cerebellum |
| ENSG00000168137 | 0.411216408 | -0.03995 | Brain_Cerebellum |
| ENSG00000270207 | 0.077668024 | -0.0191769 | Brain_Cerebellum |
| ENSG00000156959 | 0.184282158 | -0.0257281 | Brain_Cerebellum |
| ENSG00000070950 | 1.335756781 | 0.138501 | Brain_Cerebellum |
| ENSG00000071282 | 0.305407084 | -0.0356578 | Brain_Cerebellum |
| ENSG00000182533 | 1.732652242 | -0.222998 | Brain_Cerebellum |
| ENSG00000227110 | 0.084395994 | -0.0161425 | Brain_Cerebellum |
| ENSG00000125046 | 0.014746919 | -0.00460023 | Brain_Cerebellum |
| ENSG00000180914 | 0.035764419 | 0.00700636 | Brain_Cerebellum |
| ENSG00000254485 | 0.226614402 | 0.0582614 | Brain_Cerebellum |
| ENSG00000070950 | 0.13566101 | 0.0233186 | Brain_Cortex |
| ENSG00000196220 | 0.979580705 | 0.0541256 | Brain_Cortex |
| ENSG00000125046 | 0.676255085 | -0.121303 | Brain_Cortex |
| ENSG00000156959 | 0.089408142 | -0.00649428 | Brain_Cortex |
| ENSG00000270207 | 0.131196917 | 0.0322342 | Brain_Cortex |
| ENSG00000182533 | 0.031169755 | -0.00938806 | Brain_Cortex |
| ENSG00000071282 | 0.846139423 | -0.0944344 | Brain_Cortex |
| ENSG00000134077 | 1.353673728 | 0.159162 | Brain_Cortex |
| ENSG00000227110 | 0.700878573 | -0.10373 | Brain_Cortex |
| ENSG00000214041 | 0.420172976 | 0.0848978 | Brain_Cortex |
| ENSG00000235830 | 0.157351451 | 0.0352475 | Brain_Cortex |
| ENSG00000180914 | 0.268944157 | 0.0499314 | Brain_Cortex |
| ENSG00000168137 | 0.536202536 | -0.0597096 | Brain_Cortex |
| ENSG00000206573 | 0.878807968 | 0.091826 | Brain_Cortex |
| ENSG00000070950 | 0.052124366 | -0.010473 | Brain_Frontal_Cortex_BA9 |
| ENSG00000196220 | 0.408513553 | -0.0288468 | Brain_Frontal_Cortex_BA9 |
| ENSG00000125046 | 0.281876303 | 0.0699427 | Brain_Frontal_Cortex_BA9 |
| ENSG00000156959 | 0.026589023 | -0.00239628 | Brain_Frontal_Cortex_BA9 |
| ENSG00000270207 | 0.326752323 | 0.0821014 | Brain_Frontal_Cortex_BA9 |
| ENSG00000182533 | 0.14962582 | -0.0399484 | Brain_Frontal_Cortex_BA9 |
| ENSG00000071282 | 0.841972406 | 0.116759 | Brain_Frontal_Cortex_BA9 |
| ENSG00000134077 | 1.191259146 | 0.149873 | Brain_Frontal_Cortex_BA9 |
| ENSG00000227110 | 0.476096227 | 0.0852701 | Brain_Frontal_Cortex_BA9 |
| ENSG00000214041 | 0.208927907 | 0.0486619 | Brain_Frontal_Cortex_BA9 |
| ENSG00000235830 | 0.034619066 | -0.0102393 | Brain_Frontal_Cortex_BA9 |
| ENSG00000180914 | 0.40668956 | -0.0698968 | Brain_Frontal_Cortex_BA9 |
| ENSG00000168137 | 0.653482729 | -0.0647569 | Brain_Frontal_Cortex_BA9 |
| ENSG00000206573 | 0.071472548 | 0.0140591 | Brain_Frontal_Cortex_BA9 |
| ENSG00000227110 | 0.834580566 | -0.0791269 | Brain_Hippocampus |
| ENSG00000214041 | 0.082038379 | 0.0198026 | Brain_Hippocampus |
| ENSG00000206573 | 0.368303642 | -0.0701923 | Brain_Hippocampus |
| ENSG00000270207 | 0.18220994 | 0.0423387 | Brain_Hippocampus |
| ENSG00000134077 | 0.031959042 | 0.00734369 | Brain_Hippocampus |
| ENSG00000235830 | 0.368309727 | -0.094678 | Brain_Hippocampus |
| ENSG00000070950 | 0.837056423 | 0.146479 | Brain_Hippocampus |
| ENSG00000180914 | 0.104866527 | 0.0276287 | Brain_Hippocampus |
| ENSG00000071282 | 0.093219039 | 0.0193424 | Brain_Hippocampus |
| ENSG00000156959 | 0.237667359 | -0.0145194 | Brain_Hippocampus |
| ENSG00000254485 | 0.007903991 | 0.00269707 | Brain_Hippocampus |
| ENSG00000125046 | 1.001751127 | 0.169969 | Brain_Hippocampus |
| ENSG00000196220 | 1.405648339 | 0.0812551 | Brain_Hippocampus |
| ENSG00000168137 | 0.886243769 | -0.0727951 | Brain_Hippocampus |
| ENSG00000156959 | 0.313859073 | -0.0289684 | Brain_Hypothalamus |
| ENSG00000168137 | 0.885245609 | 0.0830192 | Brain_Hypothalamus |
| ENSG00000134077 | 0.13475089 | 0.0269816 | Brain_Hypothalamus |
| ENSG00000125046 | 0.812611844 | 0.145723 | Brain_Hypothalamus |
| ENSG00000071282 | 0.480493582 | -0.0712586 | Brain_Hypothalamus |
| ENSG00000196220 | 0.446047831 | 0.0325299 | Brain_Hypothalamus |
| ENSG00000206573 | 0.569489578 | 0.0928977 | Brain_Hypothalamus |
| ENSG00000270207 | 1.075207619 | 0.199382 | Brain_Hypothalamus |
| ENSG00000070950 | 0.80072764 | 0.127552 | Brain_Hypothalamus |
| ENSG00000180914 | 0.221471801 | -0.0439985 | Brain_Hypothalamus |
| ENSG00000227110 | 1.460711827 | -0.140728 | Brain_Hypothalamus |
| ENSG00000235830 | 1.019982986 | 0.174339 | Brain_Hypothalamus |
| ENSG00000182533 | 1.690091037 | 0.266264 | Brain_Hypothalamus |
| ENSG00000254485 | 1.25713667 | -0.220987 | Brain_Hypothalamus |
| ENSG00000235830 | 0.072474633 | 0.0195654 | Brain_Nucleus_accumbens_basal_ganglia |
| ENSG00000180914 | 0.502546472 | -0.0876074 | Brain_Nucleus_accumbens_basal_ganglia |
| ENSG00000156959 | 0.543615745 | 0.0365671 | Brain_Nucleus_accumbens_basal_ganglia |
| ENSG00000168137 | 0.193537148 | 0.0230002 | Brain_Nucleus_accumbens_basal_ganglia |
| ENSG00000254485 | 0.091619546 | -0.0279004 | Brain_Nucleus_accumbens_basal_ganglia |
| ENSG00000125046 | 0.095142237 | -0.0202443 | Brain_Nucleus_accumbens_basal_ganglia |
| ENSG00000182533 | 0.117993167 | 0.0322302 | Brain_Nucleus_accumbens_basal_ganglia |
| ENSG00000227110 | 0.590951575 | -0.0654709 | Brain_Nucleus_accumbens_basal_ganglia |
| ENSG00000070950 | 0.531691078 | 0.0821605 | Brain_Nucleus_accumbens_basal_ganglia |
| ENSG00000196220 | 0.629342601 | 0.0723977 | Brain_Nucleus_accumbens_basal_ganglia |
| ENSG00000206573 | 0.402868886 | -0.0511004 | Brain_Nucleus_accumbens_basal_ganglia |
| ENSG00000270207 | 0.586987066 | 0.120681 | Brain_Nucleus_accumbens_basal_ganglia |
| ENSG00000134077 | 0.522899013 | 0.0655309 | Brain_Nucleus_accumbens_basal_ganglia |
| ENSG00000071282 | 0.119995177 | 0.0184924 | Brain_Nucleus_accumbens_basal_ganglia |
| ENSG00000182533 | 0.114273629 | 0.031355 | Brain_Putamen_basal_ganglia |
| ENSG00000235830 | 0.882586173 | 0.171629 | Brain_Putamen_basal_ganglia |
| ENSG00000270207 | 0.177783523 | -0.0553123 | Brain_Putamen_basal_ganglia |
| ENSG00000156959 | 0.610355569 | 0.0337663 | Brain_Putamen_basal_ganglia |
| ENSG00000125046 | 0.311224345 | 0.0765819 | Brain_Putamen_basal_ganglia |
| ENSG00000134077 | 0.809763577 | 0.094982 | Brain_Putamen_basal_ganglia |
| ENSG00000254485 | 0.181535904 | 0.0597327 | Brain_Putamen_basal_ganglia |
| ENSG00000227110 | 0.013681153 | -0.0027534 | Brain_Putamen_basal_ganglia |
| ENSG00000196220 | 0.054016672 | 0.0108618 | Brain_Putamen_basal_ganglia |
| ENSG00000071282 | 0.025002703 | 0.00547818 | Brain_Putamen_basal_ganglia |
| ENSG00000180914 | 0.319098188 | -0.0640276 | Brain_Putamen_basal_ganglia |
| ENSG00000206573 | 0.029069497 | 0.0059362 | Brain_Putamen_basal_ganglia |
| ENSG00000070950 | 0.361523722 | 0.077997 | Brain_Putamen_basal_ganglia |
| ENSG00000168137 | 0.086742613 | -0.0102757 | Brain_Putamen_basal_ganglia |
| ENSG00000134077 | 0.522300072 | 0.0787581 | Brain_Spinal_cord_cervical_c-1 |
| ENSG00000125046 | 0.03047413 | 0.0121548 | Brain_Spinal_cord_cervical_c-1 |
| ENSG00000182533 | 0.283995821 | -0.0768598 | Brain_Spinal_cord_cervical_c-1 |
| ENSG00000235830 | 1.272435158 | 0.257373 | Brain_Spinal_cord_cervical_c-1 |
| ENSG00000270207 | 0.488373283 | 0.105616 | Brain_Spinal_cord_cervical_c-1 |
| ENSG00000254485 | 0.527628298 | -0.155876 | Brain_Spinal_cord_cervical_c-1 |
| ENSG00000156959 | 0.491503949 | 0.0728656 | Brain_Spinal_cord_cervical_c-1 |
| ENSG00000196220 | 0.058103141 | 0.0125381 | Brain_Spinal_cord_cervical_c-1 |
| ENSG00000070950 | 0.11247722 | -0.0282592 | Brain_Spinal_cord_cervical_c-1 |
| ENSG00000227110 | 0.353786503 | -0.0654769 | Brain_Spinal_cord_cervical_c-1 |
| ENSG00000168137 | 0.476507162 | 0.05927 | Brain_Spinal_cord_cervical_c-1 |
| ENSG00000180914 | 0.153969229 | 0.0506421 | Brain_Spinal_cord_cervical_c-1 |
| ENSG00000071282 | 2.310108919 | 0.237058 | Brain_Spinal_cord_cervical_c-1 |
| ENSG00000206573 | 0.349807138 | 0.0729844 | Brain_Spinal_cord_cervical_c-1 |
| ENSG00000134077 | 0.286801885 | -0.0570606 | Brain_Substantia_nigra |
| ENSG00000235830 | 1.125683466 | -0.240451 | Brain_Substantia_nigra |
| ENSG00000270207 | 0.966206447 | -0.189747 | Brain_Substantia_nigra |
| ENSG00000156959 | 0.113292746 | -0.0144238 | Brain_Substantia_nigra |
| ENSG00000125046 | 0.14114051 | -0.0490597 | Brain_Substantia_nigra |
| ENSG00000254485 | 0.78606276 | 0.201262 | Brain_Substantia_nigra |
| ENSG00000182533 | 0.141781723 | -0.0435995 | Brain_Substantia_nigra |
| ENSG00000196220 | 0.407688865 | -0.0522298 | Brain_Substantia_nigra |
| ENSG00000071282 | 0.757148117 | -0.154251 | Brain_Substantia_nigra |
| ENSG00000180914 | 0.213745604 | 0.0431212 | Brain_Substantia_nigra |
| ENSG00000206573 | 0.37334455 | 0.0718443 | Brain_Substantia_nigra |
| ENSG00000227110 | 0.201673297 | -0.0360224 | Brain_Substantia_nigra |
| ENSG00000070950 | 0.797558003 | 0.165587 | Brain_Substantia_nigra |
| ENSG00000168137 | 1.935643108 | 0.162413 | Brain_Substantia_nigra |
| ENSG00000206573 | 0.244129716 | -0.0180329 | Breast_Mammary_Tissue |
| ENSG00000180914 | 1.24251287 | -0.0677527 | Breast_Mammary_Tissue |
| ENSG00000070950 | 0.213156336 | 0.0147589 | Breast_Mammary_Tissue |
| ENSG00000254485 | 0.268893336 | -0.0402905 | Breast_Mammary_Tissue |
| ENSG00000182533 | 0.229238537 | 0.0352831 | Breast_Mammary_Tissue |
| ENSG00000156959 | 1.388224665 | 0.138187 | Breast_Mammary_Tissue |
| ENSG00000134077 | 0.236985532 | -0.0228319 | Breast_Mammary_Tissue |
| ENSG00000227110 | 0.042680582 | -0.00677128 | Breast_Mammary_Tissue |
| ENSG00000196220 | 0.485763138 | -0.0267824 | Breast_Mammary_Tissue |
| ENSG00000168137 | 0.124524906 | 0.00758479 | Breast_Mammary_Tissue |
| ENSG00000125046 | 3.715230987 | 0.197733 | Breast_Mammary_Tissue |
| ENSG00000071282 | 1.487949635 | 0.0523907 | Breast_Mammary_Tissue |
| ENSG00000196220 | 0.575054693 | -0.0448134 | Cells_Cultured_fibroblasts |
| ENSG00000070950 | 0.071885916 | -0.00349169 | Cells_Cultured_fibroblasts |
| ENSG00000071282 | 0.398011673 | 0.0156474 | Cells_Cultured_fibroblasts |
| ENSG00000180914 | 0.392929041 | 0.0315901 | Cells_Cultured_fibroblasts |
| ENSG00000134077 | 0.394026246 | 0.0241033 | Cells_Cultured_fibroblasts |
| ENSG00000206573 | 0.63753072 | 0.0371023 | Cells_Cultured_fibroblasts |
| ENSG00000156959 | 0.114000813 | -0.0165913 | Cells_Cultured_fibroblasts |
| ENSG00000227110 | 0.269794476 | 0.0354839 | Cells_Cultured_fibroblasts |
| ENSG00000254485 | 0.834916004 | -0.097397 | Cells_Cultured_fibroblasts |
| ENSG00000168137 | 0.702677286 | -0.0289402 | Cells_Cultured_fibroblasts |
| ENSG00000254485 | 0.706546839 | -0.172425 | Cells_EBV-transformed_lymphocytes |
| ENSG00000070950 | 0.539138225 | 0.0779255 | Cells_EBV-transformed_lymphocytes |
| ENSG00000196220 | 0.238222462 | 0.0639443 | Cells_EBV-transformed_lymphocytes |
| ENSG00000071282 | 0.379769181 | -0.0928293 | Cells_EBV-transformed_lymphocytes |
| ENSG00000182533 | 1.754830462 | -0.268968 | Cells_EBV-transformed_lymphocytes |
| ENSG00000125046 | 0.859699412 | -0.16634 | Cells_EBV-transformed_lymphocytes |
| ENSG00000134077 | 0.142539647 | 0.0337364 | Cells_EBV-transformed_lymphocytes |
| ENSG00000206573 | 0.711654623 | 0.104206 | Cells_EBV-transformed_lymphocytes |
| ENSG00000180914 | 0.000407559 | 7.68E-05 | Cells_EBV-transformed_lymphocytes |
| ENSG00000168137 | 0.055577541 | -0.00695051 | Cells_EBV-transformed_lymphocytes |
| ENSG00000168137 | 0.666978159 | 0.0346335 | Colon_Sigmoid |
| ENSG00000254485 | 0.307632567 | -0.0511225 | Colon_Sigmoid |
| ENSG00000206573 | 0.495485642 | -0.0355715 | Colon_Sigmoid |
| ENSG00000070950 | 0.633071128 | 0.058476 | Colon_Sigmoid |
| ENSG00000134077 | 0.293612544 | 0.0243249 | Colon_Sigmoid |
| ENSG00000071282 | 0.232508706 | 0.0252458 | Colon_Sigmoid |
| ENSG00000125046 | 0.502020463 | 0.0656059 | Colon_Sigmoid |
| ENSG00000156959 | 0.486062282 | 0.0433946 | Colon_Sigmoid |
| ENSG00000227110 | 0.060215759 | 0.00918191 | Colon_Sigmoid |
| ENSG00000182533 | 1.004835766 | 0.119314 | Colon_Sigmoid |
| ENSG00000196220 | 0.060040187 | 0.007327 | Colon_Sigmoid |
| ENSG00000180914 | 0.241053305 | 0.0360986 | Colon_Sigmoid |
| ENSG00000156959 | 0.199716746 | -0.0276981 | Colon_Transverse |
| ENSG00000134077 | 0.878138257 | -0.05653 | Colon_Transverse |
| ENSG00000206573 | 0.149154121 | 0.012641 | Colon_Transverse |
| ENSG00000071282 | 0.21117785 | 0.0202515 | Colon_Transverse |
| ENSG00000227110 | 0.310612105 | 0.0369317 | Colon_Transverse |
| ENSG00000196220 | 0.242780154 | 0.0306987 | Colon_Transverse |
| ENSG00000182533 | 0.050128633 | 0.00851069 | Colon_Transverse |
| ENSG00000125046 | 0.333795686 | 0.0357959 | Colon_Transverse |
| ENSG00000180914 | 0.565277843 | 0.0747537 | Colon_Transverse |
| ENSG00000254485 | 0.333954009 | -0.0481753 | Colon_Transverse |
| ENSG00000070950 | 0.690640286 | 0.0445877 | Colon_Transverse |
| ENSG00000168137 | 0.235424889 | -0.0153703 | Colon_Transverse |
| ENSG00000156959 | 0.939355033 | 0.0762153 | Esophagus_Gastroesophageal_Junction |
| ENSG00000254485 | 0.296336406 | 0.0559826 | Esophagus_Gastroesophageal_Junction |
| ENSG00000206573 | 0.040197113 | -0.00338607 | Esophagus_Gastroesophageal_Junction |
| ENSG00000070950 | 0.050376806 | -0.00773436 | Esophagus_Gastroesophageal_Junction |
| ENSG00000071282 | 1.273530348 | 0.0917473 | Esophagus_Gastroesophageal_Junction |
| ENSG00000180914 | 0.054366001 | 0.010982 | Esophagus_Gastroesophageal_Junction |
| ENSG00000125046 | 0.410243585 | 0.0447211 | Esophagus_Gastroesophageal_Junction |
| ENSG00000168137 | 0.680510847 | 0.0360373 | Esophagus_Gastroesophageal_Junction |
| ENSG00000196220 | 0.172598415 | 0.0261117 | Esophagus_Gastroesophageal_Junction |
| ENSG00000227110 | 0.155859704 | -0.0248196 | Esophagus_Gastroesophageal_Junction |
| ENSG00000182533 | 0.809842054 | -0.0909203 | Esophagus_Gastroesophageal_Junction |
| ENSG00000134077 | 1.117259222 | 0.063685 | Esophagus_Gastroesophageal_Junction |
| ENSG00000156959 | 0.179282425 | -0.0268417 | Esophagus_Mucosa |
| ENSG00000227110 | 1.900457471 | 0.152078 | Esophagus_Mucosa |
| ENSG00000125046 | 0.057609936 | -0.00825012 | Esophagus_Mucosa |
| ENSG00000071282 | 3.355100771 | 0.150566 | Esophagus_Mucosa |
| ENSG00000168137 | 0.113835965 | -0.00824653 | Esophagus_Mucosa |
| ENSG00000196220 | 0.71223409 | -0.0445966 | Esophagus_Mucosa |
| ENSG00000206573 | 0.119940792 | -0.00986698 | Esophagus_Mucosa |
| ENSG00000134077 | 0.532624271 | 0.0274492 | Esophagus_Mucosa |
| ENSG00000182533 | 0.336442293 | -0.0454439 | Esophagus_Mucosa |
| ENSG00000180914 | 0.038709926 | 0.00555435 | Esophagus_Mucosa |
| ENSG00000070950 | 0.208155025 | 0.0145068 | Esophagus_Mucosa |
| ENSG00000168137 | 0.116407263 | 0.00684069 | Esophagus_Muscularis |
| ENSG00000254485 | 0.129391872 | 0.0227973 | Esophagus_Muscularis |
| ENSG00000206573 | 0.111807022 | 0.00680088 | Esophagus_Muscularis |
| ENSG00000070950 | 0.063773872 | -0.00810391 | Esophagus_Muscularis |
| ENSG00000134077 | 0.601959247 | -0.0317961 | Esophagus_Muscularis |
| ENSG00000071282 | 0.595907268 | -0.0395124 | Esophagus_Muscularis |
| ENSG00000125046 | 0.511521202 | -0.0442314 | Esophagus_Muscularis |
| ENSG00000180914 | 0.14760907 | 0.0227663 | Esophagus_Muscularis |
| ENSG00000156959 | 0.807566368 | 0.0565872 | Esophagus_Muscularis |
| ENSG00000227110 | 0.625128896 | -0.0647499 | Esophagus_Muscularis |
| ENSG00000182533 | 0.017851382 | -0.00303901 | Esophagus_Muscularis |
| ENSG00000196220 | 0.109856303 | 0.0129311 | Esophagus_Muscularis |
| ENSG00000182533 | 0.816670356 | -0.0530101 | Heart_Atrial_Appendage |
| ENSG00000071282 | 1.674309915 | 0.120663 | Heart_Atrial_Appendage |
| ENSG00000168137 | 0.169285616 | -0.0103154 | Heart_Atrial_Appendage |
| ENSG00000227110 | 0.943076101 | 0.0920571 | Heart_Atrial_Appendage |
| ENSG00000134077 | 0.011403144 | -0.000906237 | Heart_Atrial_Appendage |
| ENSG00000206573 | 0.010412262 | -0.0011262 | Heart_Atrial_Appendage |
| ENSG00000180914 | 0.014149768 | 0.00274459 | Heart_Atrial_Appendage |
| ENSG00000070950 | 0.21452922 | 0.0224226 | Heart_Atrial_Appendage |
| ENSG00000235830 | 1.104683554 | 0.115385 | Heart_Atrial_Appendage |
| ENSG00000254485 | 0.163890552 | -0.0317542 | Heart_Atrial_Appendage |
| ENSG00000196220 | 1.077223177 | 0.113569 | Heart_Atrial_Appendage |
| ENSG00000125046 | 0.076607269 | -0.0113691 | Heart_Atrial_Appendage |
| ENSG00000168137 | 0.262101606 | 0.0163711 | Heart_Left_Ventricle |
| ENSG00000206573 | 0.08880296 | -0.00938237 | Heart_Left_Ventricle |
| ENSG00000182533 | 0.18349025 | -0.0149725 | Heart_Left_Ventricle |
| ENSG00000254485 | 1.18419653 | -0.125598 | Heart_Left_Ventricle |
| ENSG00000235830 | 0.640348515 | 0.0565296 | Heart_Left_Ventricle |
| ENSG00000134077 | 0.144680169 | -0.00894786 | Heart_Left_Ventricle |
| ENSG00000070950 | 0.54678107 | 0.0450289 | Heart_Left_Ventricle |
| ENSG00000180914 | 0.454035135 | 0.063304 | Heart_Left_Ventricle |
| ENSG00000196220 | 0.673133886 | 0.0422927 | Heart_Left_Ventricle |
| ENSG00000071282 | 0.113541425 | 0.0117783 | Heart_Left_Ventricle |
| ENSG00000125046 | 0.359962028 | 0.0333173 | Heart_Left_Ventricle |
| ENSG00000196220 | 0.105675628 | 0.029583 | Kidney_Cortex |
| ENSG00000227110 | 0.60739097 | 0.189816 | Kidney_Cortex |
| ENSG00000134077 | 0.005649847 | 0.00212519 | Kidney_Cortex |
| ENSG00000168137 | 1.658221342 | -0.173406 | Kidney_Cortex |
| ENSG00000254485 | 0.099852532 | 0.0493401 | Kidney_Cortex |
| ENSG00000180914 | 0.20830023 | -0.0803336 | Kidney_Cortex |
| ENSG00000071282 | 0.535586468 | 0.119699 | Kidney_Cortex |
| ENSG00000206573 | 0.169901713 | 0.0474904 | Kidney_Cortex |
| ENSG00000070950 | 1.37691929 | 0.297195 | Kidney_Cortex |
| ENSG00000125046 | 0.53396768 | 0.151785 | Kidney_Cortex |
| ENSG00000254485 | 0.131945992 | 0.0279734 | Liver |
| ENSG00000125046 | 0.237976747 | 0.052905 | Liver |
| ENSG00000134077 | 0.313705224 | -0.03061 | Liver |
| ENSG00000180914 | 0.303740617 | -0.0586762 | Liver |
| ENSG00000196220 | 0.46285742 | 0.0626486 | Liver |
| ENSG00000070950 | 0.831965343 | 0.0825565 | Liver |
| ENSG00000168137 | 1.425276729 | -0.101029 | Liver |
| ENSG00000071282 | 0.671973277 | 0.0847054 | Liver |
| ENSG00000206573 | 0.289412192 | -0.0333042 | Liver |
| ENSG00000228723 | 0.690495456 | 0.0264713 | Lung |
| ENSG00000180914 | 0.180338952 | -0.0242595 | Lung |
| ENSG00000206573 | 0.015505091 | -0.00147328 | Lung |
| ENSG00000196220 | 0.511095506 | 0.0309693 | Lung |
| ENSG00000227110 | 0.536519115 | -0.0480817 | Lung |
| ENSG00000182533 | 0.553486133 | 0.0505926 | Lung |
| ENSG00000070950 | 0.455671457 | -0.0345563 | Lung |
| ENSG00000125046 | 0.158733156 | -0.0205677 | Lung |
| ENSG00000156959 | 0.203907404 | -0.0290967 | Lung |
| ENSG00000168137 | 0.918425881 | -0.0322257 | Lung |
| ENSG00000254485 | 0.177729246 | 0.0257042 | Lung |
| ENSG00000134077 | 0.071812639 | 0.00421616 | Lung |
| ENSG00000071282 | 0.599947517 | 0.0381316 | Lung |
| ENSG00000156959 | 0.557448116 | -0.144822 | Minor_Salivary_Gland |
| ENSG00000231401 | 0.171995261 | -0.0425244 | Minor_Salivary_Gland |
| ENSG00000196220 | 0.817965022 | 0.0946321 | Minor_Salivary_Gland |
| ENSG00000254485 | 0.316344295 | -0.0929754 | Minor_Salivary_Gland |
| ENSG00000168137 | 0.522914938 | 0.0526307 | Minor_Salivary_Gland |
| ENSG00000071282 | 0.395619487 | -0.0597554 | Minor_Salivary_Gland |
| ENSG00000227110 | 0.080191025 | 0.0220669 | Minor_Salivary_Gland |
| ENSG00000235830 | 0.216002143 | -0.0503054 | Minor_Salivary_Gland |
| ENSG00000206573 | 1.667319211 | -0.121702 | Minor_Salivary_Gland |
| ENSG00000182533 | 0.58236226 | 0.104168 | Minor_Salivary_Gland |
| ENSG00000180914 | 0.772650714 | 0.170316 | Minor_Salivary_Gland |
| ENSG00000070950 | 0.669178506 | 0.144206 | Minor_Salivary_Gland |
| ENSG00000125046 | 0.040907545 | 0.0117725 | Minor_Salivary_Gland |
| ENSG00000134077 | 1.058408573 | -0.162511 | Minor_Salivary_Gland |
| ENSG00000254485 | 0.265058628 | -0.0314416 | Muscle_Skeletal |
| ENSG00000168137 | 0.049788539 | -0.00330811 | Muscle_Skeletal |
| ENSG00000180914 | 0.076476734 | 0.00833282 | Muscle_Skeletal |
| ENSG00000071282 | 0.967659309 | 0.0343987 | Muscle_Skeletal |
| ENSG00000206573 | 0.67918305 | 0.033219 | Muscle_Skeletal |
| ENSG00000125046 | 0.429053307 | -0.0456754 | Muscle_Skeletal |
| ENSG00000182533 | 0.253246737 | -0.015625 | Muscle_Skeletal |
| ENSG00000070950 | 0.294822468 | 0.0263531 | Muscle_Skeletal |
| ENSG00000134077 | 0.245825264 | 0.013485 | Muscle_Skeletal |
| ENSG00000196220 | 0.459042692 | 0.0292975 | Muscle_Skeletal |
| ENSG00000206573 | 0.555516445 | 0.0268178 | Nerve_Tibial |
| ENSG00000254485 | 0.041045475 | 0.00733734 | Nerve_Tibial |
| ENSG00000270207 | 0.340985635 | -0.0454848 | Nerve_Tibial |
| ENSG00000168137 | 0.304217224 | -0.0134416 | Nerve_Tibial |
| ENSG00000227110 | 1.264424253 | -0.073654 | Nerve_Tibial |
| ENSG00000070950 | 0.076781895 | -0.00905451 | Nerve_Tibial |
| ENSG00000182533 | 0.148054053 | -0.0202553 | Nerve_Tibial |
| ENSG00000134077 | 0.479810026 | 0.0313201 | Nerve_Tibial |
| ENSG00000180914 | 1.230870913 | -0.104239 | Nerve_Tibial |
| ENSG00000125046 | 0.073403946 | -0.0116267 | Nerve_Tibial |
| ENSG00000071282 | 0.28908251 | 0.0219074 | Nerve_Tibial |
| ENSG00000156959 | 0.209436189 | 0.0294892 | Nerve_Tibial |
| ENSG00000196220 | 0.280739167 | 0.0202109 | Nerve_Tibial |
| ENSG00000206573 | 0.550842619 | 0.0707545 | Ovary |
| ENSG00000156959 | 0.627763887 | 0.116663 | Ovary |
| ENSG00000180914 | 0.077218508 | 0.0224522 | Ovary |
| ENSG00000134077 | 0.360021722 | -0.0430003 | Ovary |
| ENSG00000196220 | 0.092582189 | -0.0161933 | Ovary |
| ENSG00000182533 | 0.030195633 | -0.00787422 | Ovary |
| ENSG00000071282 | 0.473119484 | 0.0730895 | Ovary |
| ENSG00000125046 | 0.173206937 | 0.0457139 | Ovary |
| ENSG00000070950 | 0.17502204 | -0.0340674 | Ovary |
| ENSG00000227110 | 0.477679682 | 0.0692446 | Ovary |
| ENSG00000168137 | 0.105590884 | 0.0154179 | Ovary |
| ENSG00000125046 | 0.089006548 | -0.0185599 | Pancreas |
| ENSG00000071282 | 0.124871571 | 0.0197668 | Pancreas |
| ENSG00000134077 | 0.532411131 | 0.0545749 | Pancreas |
| ENSG00000196220 | 0.043450768 | 0.006428 | Pancreas |
| ENSG00000156959 | 0.115215961 | -0.013603 | Pancreas |
| ENSG00000254485 | 0.157446299 | 0.0337177 | Pancreas |
| ENSG00000168137 | 0.225011041 | -0.0188616 | Pancreas |
| ENSG00000070950 | 0.281558103 | -0.0451656 | Pancreas |
| ENSG00000180914 | 0.347343739 | -0.0530134 | Pancreas |
| ENSG00000206573 | 0.412622459 | 0.0450413 | Pancreas |
| ENSG00000227110 | 0.411386596 | 0.0830562 | Pituitary |
| ENSG00000228723 | 0.290353587 | -0.0489233 | Pituitary |
| ENSG00000070950 | 1.656280322 | 0.131991 | Pituitary |
| ENSG00000254485 | 0.270359853 | -0.0639586 | Pituitary |
| ENSG00000180914 | 0.231991231 | 0.0374399 | Pituitary |
| ENSG00000125046 | 0.331030136 | 0.054209 | Pituitary |
| ENSG00000156959 | 0.570902076 | -0.0454448 | Pituitary |
| ENSG00000196220 | 0.459207691 | -0.052641 | Pituitary |
| ENSG00000235830 | 0.592057017 | 0.106699 | Pituitary |
| ENSG00000168137 | 0.050108161 | 0.00571237 | Pituitary |
| ENSG00000071282 | 0.09618372 | -0.0148907 | Pituitary |
| ENSG00000206573 | 0.531636421 | 0.0660454 | Pituitary |
| ENSG00000134077 | 0.370654632 | -0.050452 | Pituitary |
| ENSG00000254485 | 0.267553163 | 0.0496457 | Prostate |
| ENSG00000206573 | 0.057371473 | -0.00760126 | Prostate |
| ENSG00000182533 | 0.335911115 | -0.0550244 | Prostate |
| ENSG00000227110 | 0.50848703 | -0.0717388 | Prostate |
| ENSG00000071282 | 0.195649267 | -0.0314015 | Prostate |
| ENSG00000070950 | 0.204067176 | -0.0301952 | Prostate |
| ENSG00000196220 | 0.623158532 | -0.0544698 | Prostate |
| ENSG00000125046 | 0.960982678 | -0.136471 | Prostate |
| ENSG00000168137 | 0.064522461 | 0.00711512 | Prostate |
| ENSG00000180914 | 0.327559462 | -0.0623112 | Prostate |
| ENSG00000134077 | 0.772679017 | 0.0684718 | Prostate |
| ENSG00000156959 | 1.016226267 | 0.169988 | Prostate |
| ENSG00000125046 | 0.580244106 | -0.0547048 | Skin_Not_Sun_Exposed_Suprapubic |
| ENSG00000168137 | 0.339203749 | -0.0146557 | Skin_Not_Sun_Exposed_Suprapubic |
| ENSG00000254485 | 0.099307955 | 0.0157372 | Skin_Not_Sun_Exposed_Suprapubic |
| ENSG00000134077 | 0.583825653 | 0.036349 | Skin_Not_Sun_Exposed_Suprapubic |
| ENSG00000196220 | 0.111192273 | -0.00950443 | Skin_Not_Sun_Exposed_Suprapubic |
| ENSG00000070950 | 0.358197814 | 0.0264541 | Skin_Not_Sun_Exposed_Suprapubic |
| ENSG00000182533 | 0.102878964 | 0.0157637 | Skin_Not_Sun_Exposed_Suprapubic |
| ENSG00000206573 | 0.061006712 | -0.00404428 | Skin_Not_Sun_Exposed_Suprapubic |
| ENSG00000071282 | 0.220718884 | 0.0142744 | Skin_Not_Sun_Exposed_Suprapubic |
| ENSG00000156959 | 0.436632843 | -0.0579711 | Skin_Not_Sun_Exposed_Suprapubic |
| ENSG00000227110 | 0.240249081 | -0.0279509 | Skin_Not_Sun_Exposed_Suprapubic |
| ENSG00000180914 | 0.056561877 | -0.00834189 | Skin_Not_Sun_Exposed_Suprapubic |
| ENSG00000070950 | 0.271146458 | -0.0206729 | Skin_Sun_Exposed_Lower_leg |
| ENSG00000182533 | 0.27863627 | 0.0332846 | Skin_Sun_Exposed_Lower_leg |
| ENSG00000180914 | 0.010383794 | -0.00155849 | Skin_Sun_Exposed_Lower_leg |
| ENSG00000206573 | 0.001990147 | 0.000128523 | Skin_Sun_Exposed_Lower_leg |
| ENSG00000156959 | 0.155039095 | 0.0210783 | Skin_Sun_Exposed_Lower_leg |
| ENSG00000134077 | 0.731864504 | 0.0376324 | Skin_Sun_Exposed_Lower_leg |
| ENSG00000196220 | 0.587164962 | -0.03221 | Skin_Sun_Exposed_Lower_leg |
| ENSG00000125046 | 0.064562267 | -0.00804613 | Skin_Sun_Exposed_Lower_leg |
| ENSG00000227110 | 0.859998196 | -0.073869 | Skin_Sun_Exposed_Lower_leg |
| ENSG00000071282 | 1.234113235 | -0.0479032 | Skin_Sun_Exposed_Lower_leg |
| ENSG00000168137 | 0.07878292 | 0.0044676 | Skin_Sun_Exposed_Lower_leg |
| ENSG00000254485 | 0.0785747 | 0.0123798 | Skin_Sun_Exposed_Lower_leg |
| ENSG00000156959 | 0.080769147 | -0.0147869 | Small_Intestine_Terminal_Ileum |
| ENSG00000125046 | 0.033061306 | -0.00518791 | Small_Intestine_Terminal_Ileum |
| ENSG00000180914 | 0.054190809 | -0.0135749 | Small_Intestine_Terminal_Ileum |
| ENSG00000070950 | 0.059863191 | -0.0110234 | Small_Intestine_Terminal_Ileum |
| ENSG00000182533 | 0.17184041 | -0.0338017 | Small_Intestine_Terminal_Ileum |
| ENSG00000254485 | 0.015973426 | 0.00545432 | Small_Intestine_Terminal_Ileum |
| ENSG00000206573 | 0.364257965 | -0.0405977 | Small_Intestine_Terminal_Ileum |
| ENSG00000196220 | 0.378168615 | -0.0708215 | Small_Intestine_Terminal_Ileum |
| ENSG00000168137 | 0.01084441 | 0.00145093 | Small_Intestine_Terminal_Ileum |
| ENSG00000134077 | 0.323177493 | 0.0458703 | Small_Intestine_Terminal_Ileum |
| ENSG00000071282 | 0.547896016 | -0.0624231 | Small_Intestine_Terminal_Ileum |
| ENSG00000134077 | 0.545728482 | 0.0694713 | Spleen |
| ENSG00000168137 | 0.004557429 | -0.000528129 | Spleen |
| ENSG00000182533 | 0.811577785 | -0.122416 | Spleen |
| ENSG00000180914 | 1.216849231 | -0.160725 | Spleen |
| ENSG00000206573 | 0.610872915 | -0.057175 | Spleen |
| ENSG00000227110 | 0.015239625 | -0.00375417 | Spleen |
| ENSG00000196220 | 0.374040706 | 0.0632698 | Spleen |
| ENSG00000254485 | 0.131230404 | -0.0335809 | Spleen |
| ENSG00000070950 | 0.038603588 | -0.00693205 | Spleen |
| ENSG00000125046 | 2.678362222 | -0.242756 | Spleen |
| ENSG00000071282 | 0.419579144 | 0.0435412 | Spleen |
| ENSG00000070950 | 0.261638881 | 0.0335871 | Stomach |
| ENSG00000227110 | 0.235132238 | 0.0433723 | Stomach |
| ENSG00000134077 | 0.262715099 | 0.0267907 | Stomach |
| ENSG00000180914 | 0.206338107 | 0.0348215 | Stomach |
| ENSG00000182533 | 0.341556433 | 0.0602176 | Stomach |
| ENSG00000196220 | 0.716253396 | 0.0641905 | Stomach |
| ENSG00000254485 | 0.529590979 | -0.0789781 | Stomach |
| ENSG00000168137 | 0.220356619 | 0.0180636 | Stomach |
| ENSG00000071282 | 0.194159451 | -0.0205498 | Stomach |
| ENSG00000125046 | 0.480068365 | 0.0676552 | Stomach |
| ENSG00000156959 | 0.094338475 | 0.0158139 | Stomach |
| ENSG00000206573 | 0.411138054 | 0.0367143 | Stomach |
| ENSG00000254485 | 0.161933974 | -0.0261363 | Testis |
| ENSG00000206573 | 0.034835001 | 0.00353776 | Testis |
| ENSG00000227929 | 0.163181096 | -0.0162176 | Testis |
| ENSG00000196220 | 1.024975102 | 0.0720377 | Testis |
| ENSG00000180914 | 0.185057556 | -0.0297908 | Testis |
| ENSG00000228351 | 0.440720743 | -0.0687226 | Testis |
| ENSG00000182533 | 0.23157651 | 0.0315683 | Testis |
| ENSG00000224808 | 0.147267555 | -0.0289054 | Testis |
| ENSG00000270207 | 0.24102607 | 0.0489085 | Testis |
| ENSG00000156959 | 0.750919102 | 0.0729514 | Testis |
| ENSG00000228723 | 0.07252082 | -0.0170786 | Testis |
| ENSG00000231401 | 0.760362729 | -0.102226 | Testis |
| ENSG00000070950 | 0.046228231 | 0.00575645 | Testis |
| ENSG00000227110 | 0.039054279 | -0.00909134 | Testis |
| ENSG00000134077 | 0.264838009 | -0.0146543 | Testis |
| ENSG00000168137 | 0.385728323 | -0.0167077 | Testis |
| ENSG00000125046 | 0.713235995 | -0.031436 | Testis |
| ENSG00000215160 | 0.011393336 | -0.0020726 | Testis |
| ENSG00000071282 | 1.404545666 | 0.0677319 | Testis |
| ENSG00000214041 | 2.065156775 | -0.129995 | Thyroid |
| ENSG00000196220 | 0.55713941 | 0.0370203 | Thyroid |
| ENSG00000168137 | 0.260570071 | 0.0132612 | Thyroid |
| ENSG00000206573 | 0.322378449 | -0.0167636 | Thyroid |
| ENSG00000254485 | 1.849268115 | -0.107777 | Thyroid |
| ENSG00000227110 | 0.29302206 | 0.033123 | Thyroid |
| ENSG00000182533 | 1.064397522 | 0.0852965 | Thyroid |
| ENSG00000180914 | 0.235253903 | 0.0270368 | Thyroid |
| ENSG00000070950 | 0.489927072 | 0.0321921 | Thyroid |
| ENSG00000235830 | 0.399395159 | 0.0410821 | Thyroid |
| ENSG00000071282 | 0.216678966 | -0.0197319 | Thyroid |
| ENSG00000156959 | 1.098032485 | 0.100437 | Thyroid |
| ENSG00000134077 | 0.345686739 | 0.0179833 | Thyroid |
| ENSG00000125046 | 0.041456184 | 0.00485015 | Thyroid |
| ENSG00000071282 | 0.232720174 | 0.0394505 | Uterus |
| ENSG00000206573 | 1.313357476 | 0.145539 | Uterus |
| ENSG00000196220 | 0.67796894 | 0.111638 | Uterus |
| ENSG00000125046 | 0.459784433 | 0.105215 | Uterus |
| ENSG00000182533 | 0.14909045 | -0.0454123 | Uterus |
| ENSG00000070950 | 0.25182283 | -0.0631834 | Uterus |
| ENSG00000156959 | 0.087180867 | 0.0311659 | Uterus |
| ENSG00000134077 | 0.356041271 | -0.0547434 | Uterus |
| ENSG00000168137 | 0.099345622 | 0.014586 | Uterus |
| ENSG00000180914 | 1.041249348 | 0.217738 | Uterus |
| ENSG00000227110 | 0.293026324 | -0.0783087 | Uterus |
| ENSG00000206573 | 0.998417711 | 0.142916 | Vagina |
| ENSG00000156959 | 1.272768715 | 0.239879 | Vagina |
| ENSG00000125046 | 0.32365305 | 0.0787394 | Vagina |
| ENSG00000180914 | 0.055444787 | 0.0169977 | Vagina |
| ENSG00000168137 | 0.240088264 | 0.0299796 | Vagina |
| ENSG00000134077 | 1.258466296 | 0.11548 | Vagina |
| ENSG00000227110 | 0.239444836 | -0.0509714 | Vagina |
| ENSG00000182533 | 0.038382924 | 0.0115796 | Vagina |
| ENSG00000070950 | 0.012371268 | 0.00338686 | Vagina |
| ENSG00000071282 | 0.028224721 | 0.00486916 | Vagina |
| ENSG00000196220 | 1.084468788 | -0.158702 | Vagina |
| ENSG00000070950 | 0.007408047 | 0.000623268 | Whole_Blood |
| ENSG00000206573 | 0.153817579 | -0.0097229 | Whole_Blood |
| ENSG00000180914 | 0.289078285 | -0.0347778 | Whole_Blood |
| ENSG00000134077 | 0.510951822 | -0.0311019 | Whole_Blood |
| ENSG00000071282 | 0.290863232 | -0.0212628 | Whole_Blood |
| ENSG00000196220 | 1.336590272 | 0.0846655 | Whole_Blood |
| ENSG00000168137 | 0.316382987 | 0.00945392 | Whole_Blood |
| ENSG00000070950 | 0.29728003 | 0.00964873 | iPSC |
| ENSG00000071282 | 0.395244905 | 0.0328052 | iPSC |
| ENSG00000134077 | 0.341245689 | -0.0126378 | iPSC |
| ENSG00000156959 | 0.055806625 | -0.00539961 | iPSC |
| ENSG00000168137 | 0.047896099 | 0.00206581 | iPSC |
| ENSG00000180914 | 0.66167501 | -0.0593938 | iPSC |
| ENSG00000196220 | 0.069449297 | -0.00540355 | iPSC |
| ENSG00000206573 | 0.146452053 | 0.00980867 | iPSC |
| ENSG00000224884 | 0.699798124 | 0.105347 | iPSC |
| ENSG00000227110 | 0.628339315 | 0.0547146 | iPSC |
| ENSG00000254485 | 0.214603958 | 0.0393517 | iPSC |
| ENSG00000070950 | 0.479312141 | -0.0123931 | blood |
| ENSG00000134077 | 0.156746037 | -0.00411355 | blood |
| ENSG00000168137 | 0.184037268 | -0.00296002 | blood |
| ENSG00000180914 | 0.711905108 | -0.0581449 | blood |
| ENSG00000206573 | 0.219966548 | 0.00748259 | blood |
| ENSG00000070950 | 0.255879987 | -0.0222747 | macrophage_Listeria |
| ENSG00000071282 | 0.665208596 | -0.164438 | macrophage_Listeria |
| ENSG00000134077 | 0.247790931 | -0.0143886 | macrophage_Listeria |
| ENSG00000168137 | 0.314015662 | -0.0160411 | macrophage_Listeria |
| ENSG00000180914 | 0.010642302 | 0.00379468 | macrophage_Listeria |
| ENSG00000196220 | 0.739108523 | -0.0715092 | macrophage_Listeria |
| ENSG00000206573 | 0.240156207 | -0.0265854 | macrophage_Listeria |
| ENSG00000196220 | 0.490102893 | -0.0444846 | macrophage_Salmonella |
| ENSG00000070950 | 0.089640841 | -0.00917338 | monocyte_IAV |
| ENSG00000134077 | 0.013467865 | -0.001549 | monocyte_IAV |
| ENSG00000156959 | 0.096735245 | -0.017906 | monocyte_IAV |
| ENSG00000168137 | 0.324998402 | -0.0177523 | monocyte_IAV |
| ENSG00000206573 | 0.328170194 | -0.0277287 | monocyte_IAV |
| ENSG00000254485 | 0.219357269 | -0.0462841 | monocyte_IAV |
| ENSG00000070950 | 0.3717562 | -0.034596 | monocyte_LPS |
| ENSG00000134077 | 0.537215802 | 0.0330555 | monocyte_LPS |
| ENSG00000168137 | 0.03070759 | -0.00198657 | monocyte_LPS |
| ENSG00000196220 | 0.202092917 | 0.0393976 | monocyte_LPS |
| ENSG00000206573 | 0.335746431 | 0.0316271 | monocyte_LPS |
| ENSG00000070950 | 0.331472448 | -0.0189746 | monocyte_naive |
| ENSG00000134077 | 0.49438205 | 0.0286518 | monocyte_naive |
| ENSG00000168137 | 0.762659885 | 0.035009 | monocyte_naive |
| ENSG00000206573 | 0.029599654 | 0.00335705 | monocyte_naive |
| ENSG00000070950 | 0.217023142 | -0.0199057 | monocyte_Pam3CSK4 |
| ENSG00000134077 | 0.045722266 | 0.00385339 | monocyte_Pam3CSK4 |
| ENSG00000168137 | 0.306739245 | 0.0154812 | monocyte_Pam3CSK4 |
| ENSG00000196220 | 0.894132308 | 0.101691 | monocyte_Pam3CSK4 |
| ENSG00000206573 | 0.169447257 | 0.0173417 | monocyte_Pam3CSK4 |
| ENSG00000070950 | 0.016081576 | 0.00228105 | monocyte_R848 |
| ENSG00000134077 | 0.365643664 | -0.0237169 | monocyte_R848 |
| ENSG00000168137 | 0.493886144 | -0.023255 | monocyte_R848 |
| ENSG00000196220 | 0.612147647 | 0.0923032 | monocyte_R848 |
| ENSG00000206573 | 0.223601766 | -0.0206377 | monocyte_R848 |
| ENSG00000070950 | 0.284851052 | -0.0100013 | brain_naive |
| ENSG00000071282 | 1.159098846 | -0.0500874 | brain_naive |
| ENSG00000125046 | 0.004228835 | 0.00025621 | brain_naive |
| ENSG00000134077 | 0.84520489 | 0.0138647 | brain_naive |
| ENSG00000156959 | 0.194764285 | -0.00453529 | brain_naive |
| ENSG00000168137 | 0.678163467 | 0.00980664 | brain_naive |
| ENSG00000180914 | 0.761918753 | 0.0634975 | brain_naive |
| ENSG00000196220 | 0.387294535 | -0.00618884 | brain_naive |
| ENSG00000206573 | 0.802245424 | -0.0204834 | brain_naive |
| ENSG00000227110 | 0.266604498 | -0.0131314 | brain_naive |
| ENSG00000231401 | 0.400347231 | 0.0246775 | brain_naive |
| ENSG00000235830 | 0.399879113 | 0.0289582 | brain_naive |
| ENSG00000254485 | 0.779248795 | 0.0392435 | brain_naive |
| ENSG00000270207 | 0.234224933 | 0.0142729 | brain_naive |
| ENSG00000070950 | 0.025075394 | 0.00141004 | B-cell_naive |
| ENSG00000134077 | 0.221436366 | -0.0127025 | B-cell_naive |
| ENSG00000168137 | 0.059242529 | -0.00234835 | B-cell_naive |
| ENSG00000206573 | 0.733251034 | -0.0404936 | B-cell_naive |
| ENSG00000254485 | 0.271438456 | 0.09508 | B-cell_naive |
| ENSG00000070950 | 0.570149248 | 0.0202612 | CD4_T-cell_anti-CD3-CD28 |
| ENSG00000134077 | 0.585035004 | -0.0184895 | CD4_T-cell_anti-CD3-CD28 |
| ENSG00000168137 | 0.32777834 | 0.00701716 | CD4_T-cell_anti-CD3-CD28 |
| ENSG00000206573 | 0.366440655 | -0.0205407 | CD4_T-cell_anti-CD3-CD28 |
| ENSG00000070950 | 0.327152476 | 0.0148643 | CD4_T-cell_naive |
| ENSG00000134077 | 0.466187284 | -0.0188703 | CD4_T-cell_naive |
| ENSG00000168137 | 0.733714196 | -0.0170183 | CD4_T-cell_naive |
| ENSG00000180914 | 0.681404563 | -0.167658 | CD4_T-cell_naive |
| ENSG00000196220 | 0.158677452 | -0.0241645 | CD4_T-cell_naive |
| ENSG00000206573 | 0.360888202 | -0.0252379 | CD4_T-cell_naive |
| ENSG00000254485 | 0.366154983 | 0.121921 | CD4_T-cell_naive |
| ENSG00000070950 | 0.869640496 | 0.0271806 | CD8_T-cell_anti-CD3-CD28 |
| ENSG00000134077 | 0.48147948 | -0.0148332 | CD8_T-cell_anti-CD3-CD28 |
| ENSG00000168137 | 0.055083743 | -0.00167459 | CD8_T-cell_anti-CD3-CD28 |
| ENSG00000206573 | 0.037000755 | -0.0029512 | CD8_T-cell_anti-CD3-CD28 |
| ENSG00000070950 | 0.579197955 | -0.0218044 | CD8_T-cell_naive |
| ENSG00000134077 | 0.685852476 | -0.0235155 | CD8_T-cell_naive |
| ENSG00000168137 | 0.345797459 | -0.00809407 | CD8_T-cell_naive |
| ENSG00000180914 | 0.444505314 | 0.111353 | CD8_T-cell_naive |
| ENSG00000196220 | 0.870364881 | -0.120498 | CD8_T-cell_naive |
| ENSG00000206573 | 0.906501269 | -0.0426361 | CD8_T-cell_naive |
| ENSG00000235830 | 0.279138122 | 0.0539655 | CD8_T-cell_naive |
| ENSG00000254485 | 0.650419447 | 0.193441 | CD8_T-cell_naive |
| ENSG00000070950 | 0.314144554 | -0.0132459 | monocyte_CD16_naive |
| ENSG00000134077 | 0.36128916 | 0.0132925 | monocyte_CD16_naive |
| ENSG00000168137 | 0.458267283 | 0.0111888 | monocyte_CD16_naive |
| ENSG00000206573 | 1.714442691 | -0.06794 | monocyte_CD16_naive |
| ENSG00000196220 | 0.019397216 | -0.00231535 | monocyte_naive |
| ENSG00000270207 | 0.162258197 | -0.0476765 | monocyte_naive |
| ENSG00000070950 | 0.139563764 | -0.00750904 | NK-cell_naive |
| ENSG00000134077 | 0.023252596 | -0.00147162 | NK-cell_naive |
| ENSG00000168137 | 0.160459735 | 0.00666404 | NK-cell_naive |
| ENSG00000180914 | 0.491619785 | -0.148632 | NK-cell_naive |
| ENSG00000206573 | 0.300871073 | -0.0231161 | NK-cell_naive |
| ENSG00000070950 | 0.192575927 | 0.00944401 | Tfh_memory |
| ENSG00000134077 | 1.249484663 | 0.0405245 | Tfh_memory |
| ENSG00000168137 | 0.198817267 | -0.00421035 | Tfh_memory |
| ENSG00000180914 | 1.784312217 | -0.276361 | Tfh_memory |
| ENSG00000196220 | 0.516549645 | 0.0959075 | Tfh_memory |
| ENSG00000206573 | 0.858575313 | -0.0433758 | Tfh_memory |
| ENSG00000070950 | 0.101292366 | 0.00465135 | Th1-17_memory |
| ENSG00000134077 | 0.46813167 | 0.0195875 | Th1-17_memory |
| ENSG00000168137 | 0.705350836 | -0.0141608 | Th1-17_memory |
| ENSG00000180914 | 0.605924988 | -0.124593 | Th1-17_memory |
| ENSG00000206573 | 0.007266705 | -0.000603215 | Th1-17_memory |
| ENSG00000070950 | 0.095076281 | -0.00377619 | Th17_memory |
| ENSG00000071282 | 0.795852875 | -0.156363 | Th17_memory |
| ENSG00000134077 | 0.332803964 | 0.0161691 | Th17_memory |
| ENSG00000168137 | 0.170799183 | -0.00465469 | Th17_memory |
| ENSG00000180914 | 0.043788815 | -0.0114826 | Th17_memory |
| ENSG00000206573 | 0.295560336 | -0.0197799 | Th17_memory |
| ENSG00000070950 | 0.361566656 | -0.0169314 | Th1_memory |
| ENSG00000134077 | 0.205247135 | 0.0105799 | Th1_memory |
| ENSG00000168137 | 0.085263458 | -0.00270461 | Th1_memory |
| ENSG00000180914 | 0.208565513 | -0.0533961 | Th1_memory |
| ENSG00000196220 | 0.841902991 | 0.109552 | Th1_memory |
| ENSG00000206573 | 0.903138631 | -0.0458596 | Th1_memory |
| ENSG00000070950 | 0.011668059 | 0.000700234 | Th2_memory |
| ENSG00000071282 | 1.915552421 | -0.334097 | Th2_memory |
| ENSG00000134077 | 0.48991097 | 0.021482 | Th2_memory |
| ENSG00000168137 | 0.39068098 | -0.0105989 | Th2_memory |
| ENSG00000180914 | 0.827732843 | -0.17377 | Th2_memory |
| ENSG00000196220 | 0.139315295 | 0.0333852 | Th2_memory |
| ENSG00000206573 | 1.052062671 | -0.0514659 | Th2_memory |
| ENSG00000070950 | 0.009989432 | -0.000603453 | Treg_memory |
| ENSG00000071282 | 2.034048459 | -0.123577 | Treg_memory |
| ENSG00000134077 | 0.510808186 | 0.0227565 | Treg_memory |
| ENSG00000168137 | 1.11081263 | 0.0229427 | Treg_memory |
| ENSG00000180914 | 2.24177717 | -0.31924 | Treg_memory |
| ENSG00000196220 | 0.395211457 | -0.040425 | Treg_memory |
| ENSG00000206573 | 0.743799579 | -0.0385799 | Treg_memory |
| ENSG00000070950 | 0.283827148 | -0.0121952 | Treg_naive |
| ENSG00000071282 | 6.944805212 | -0.665755 | Treg_naive |
| ENSG00000134077 | 0.013304386 | 0.000884943 | Treg_naive |
| ENSG00000168137 | 0.589210961 | 0.0142082 | Treg_naive |
| ENSG00000180914 | 0.790672684 | -0.212116 | Treg_naive |
| ENSG00000196220 | 0.016003165 | 0.00318792 | Treg_naive |
| ENSG00000206573 | 1.458617981 | -0.0678389 | Treg_naive |
| ENSG00000254485 | 0.075583209 | -0.0304418 | Treg_naive |
| ENSG00000070950 | 0.07310269 | 0.0109819 | sensory_neuron |
| ENSG00000071282 | 0.440526689 | -0.129105 | sensory_neuron |
| ENSG00000125046 | 0.253063146 | -0.133791 | sensory_neuron |
| ENSG00000134077 | 0.238335222 | -0.03646 | sensory_neuron |
| ENSG00000156959 | 1.710440031 | 0.130446 | sensory_neuron |
| ENSG00000168137 | 0.074691519 | 0.00733248 | sensory_neuron |
| ENSG00000180914 | 0.499098318 | 0.156171 | sensory_neuron |
| ENSG00000196220 | 0.350572644 | -0.0505696 | sensory_neuron |
| ENSG00000206573 | 0.077629075 | -0.0109439 | sensory_neuron |
| ENSG00000227110 | 1.099972245 | -0.182203 | sensory_neuron |
| ENSG00000231401 | 0.590401598 | -0.248387 | sensory_neuron |
| ENSG00000235830 | 0.240235488 | 0.0672226 | sensory_neuron |
| ENSG00000070950 | 0.449717822 | 0.0158739 | fat |
| ENSG00000071282 | 0.109027102 | 0.00705933 | fat |
| ENSG00000125046 | 0.25369437 | -0.0317821 | fat |
| ENSG00000134077 | 0.259713911 | -0.0062547 | fat |
| ENSG00000168137 | 0.819509975 | 0.0132401 | fat |
| ENSG00000196220 | 0.081467997 | 0.00689207 | fat |
| ENSG00000206573 | 1.205784606 | -0.0280883 | fat |
| ENSG00000235830 | 0.187968822 | 0.0114213 | fat |
| ENSG00000254485 | 1.041116128 | 0.0843941 | fat |
| ENSG00000182533 | 0.192243822 | 0.0280564 | LCL |
| ENSG00000235830 | 0.022657369 | -0.00181245 | LCL |
| ENSG00000070950 | 0.492191337 | 0.0197765 | skin |
| ENSG00000071282 | 0.588121141 | -0.027366 | skin |
| ENSG00000125046 | 0.019441723 | 0.00228179 | skin |
| ENSG00000134077 | 0.000120316 | 5.08E-06 | skin |
| ENSG00000156959 | 0.00771253 | 0.000909256 | skin |
| ENSG00000168137 | 0.243481038 | -0.00638934 | skin |
| ENSG00000180914 | 0.927364153 | -0.0623527 | skin |
| ENSG00000182533 | 0.479525638 | -0.0488241 | skin |
| ENSG00000196220 | 0.087411845 | -0.00644336 | skin |
| ENSG00000206573 | 0.022597892 | -0.00129209 | skin |
| ENSG00000227929 | 0.056373435 | -0.00511351 | skin |
| ENSG00000235830 | 0.111317959 | -0.0148221 | skin |
| ENSG00000254485 | 0.637981473 | 0.0517954 | skin |
| ENSG00000070950 | 0.158123314 | 0.0288125 | pancreatic_islet |
| ENSG00000071282 | 0.962880406 | 0.134242 | pancreatic_islet |
| ENSG00000125046 | 0.155013029 | -0.041609 | pancreatic_islet |
| ENSG00000134077 | 0.450886338 | 0.025507 | pancreatic_islet |
| ENSG00000156959 | 0.072009439 | -0.0137846 | pancreatic_islet |
| ENSG00000168137 | 0.024220895 | -0.00211394 | pancreatic_islet |
| ENSG00000180914 | 0.264917131 | -0.0733657 | pancreatic_islet |
| ENSG00000196220 | 1.598299693 | 0.181362 | pancreatic_islet |
| ENSG00000206573 | 0.237421208 | 0.0280443 | pancreatic_islet |
| ENSG00000224808 | 0.09185397 | -0.0219904 | pancreatic_islet |
| ENSG00000224884 | 0.435601458 | 0.143765 | pancreatic_islet |
| ENSG00000227110 | 0.028286829 | -0.00740284 | pancreatic_islet |
| ENSG00000227929 | 2.817813683 | 0.305231 | pancreatic_islet |
| ENSG00000228723 | 0.474372204 | 0.150545 | pancreatic_islet |
| ENSG00000231401 | 0.17812831 | 0.0523523 | pancreatic_islet |
| ENSG00000235830 | 0.719073514 | 0.16662 | pancreatic_islet |
| ENSG00000254485 | 0.148517833 | 0.0334145 | pancreatic_islet |
