# Supplementary material for: Linc00312 Single Nucleotide Polymorphism as Biomarker for Chemoradiotherapy Induced Hematotoxicity in Nasopharyngeal Carcinoma Patients
Source: Dis Markers. 2022 Aug 8;2022:6707821. doi: 10.1155/2022/6707821 (PMC9381851; doi:10.1155/2022/6707821)
Supplement: Supplementary 4 — Supplementary Table 4: data from Ensembl indicates the gene expression correlated with rs15734. [file 6707821.f4.docx]

| **Supplementary Table 4. Data from Ensembl indicates the gene expression correlated with rs15734.** | | | |
| --- | --- | --- | --- |
| **Gene** | **P-value (-log10)** | **Effect size** | **Tissue** |
| ENSG00000071282 | 0.075931708 | -0.00682871 | adipose_naive |
| ENSG00000182533 | 0.097977517 | -0.0335381 | adipose_naive |
| ENSG00000125046 | 0.423499706 | -0.0685193 | adipose_naive |
| ENSG00000134077 | 0.488774592 | 0.015585 | adipose_naive |
| ENSG00000070950 | 0.664345558 | 0.0241478 | adipose_naive |
| ENSG00000206573 | 0.670380453 | 0.0276426 | adipose_naive |
| ENSG00000196220 | 0.712410992 | 0.0480384 | adipose_naive |
| ENSG00000168137 | 1.088596805 | 0.0258015 | adipose_naive |
| ENSG00000134077 | 0.018834457 | 0.00184495 | Adipose_Subcutaneous |
| ENSG00000182533 | 0.057519196 | -0.00831083 | Adipose_Subcutaneous |
| ENSG00000168137 | 0.081059543 | 0.00562867 | Adipose_Subcutaneous |
| ENSG00000070950 | 0.107114366 | 0.0116006 | Adipose_Subcutaneous |
| ENSG00000071282 | 0.16129318 | -0.0137631 | Adipose_Subcutaneous |
| ENSG00000125046 | 0.472466763 | -0.0407588 | Adipose_Subcutaneous |
| ENSG00000196220 | 0.794757732 | -0.0594165 | Adipose_Subcutaneous |
| ENSG00000180914 | 0.913266552 | 0.0911783 | Adipose_Subcutaneous |
| ENSG00000206573 | 1.270130863 | 0.0647735 | Adipose_Subcutaneous |
| ENSG00000227110 | 2.338990413 | 0.151938 | Adipose_Subcutaneous |
| ENSG00000125046 | 0.01378694 | 0.00178306 | Adipose_Visceral_Omentum |
| ENSG00000180914 | 0.066973065 | 0.0116111 | Adipose_Visceral_Omentum |
| ENSG00000182533 | 0.248683936 | -0.0293229 | Adipose_Visceral_Omentum |
| ENSG00000156959 | 0.265730766 | 0.0364658 | Adipose_Visceral_Omentum |
| ENSG00000196220 | 0.293358162 | 0.0320169 | Adipose_Visceral_Omentum |
| ENSG00000134077 | 0.417134729 | -0.0336925 | Adipose_Visceral_Omentum |
| ENSG00000168137 | 0.538498472 | -0.0314729 | Adipose_Visceral_Omentum |
| ENSG00000070950 | 0.553638388 | -0.049418 | Adipose_Visceral_Omentum |
| ENSG00000206573 | 1.423102602 | 0.087886 | Adipose_Visceral_Omentum |
| ENSG00000227110 | 3.820445337 | 0.214681 | Adipose_Visceral_Omentum |
| ENSG00000071282 | 5.191286806 | 0.146434 | Adipose_Visceral_Omentum |
| ENSG00000125046 | 0.017893468 | -0.00443002 | Adrenal_Gland |
| ENSG00000156959 | 0.065142142 | -0.0118991 | Adrenal_Gland |
| ENSG00000196220 | 0.08369716 | -0.0182792 | Adrenal_Gland |
| ENSG00000228723 | 0.236822921 | -0.046344 | Adrenal_Gland |
| ENSG00000235830 | 0.271097 | 0.0638803 | Adrenal_Gland |
| ENSG00000180914 | 0.310238432 | 0.0598562 | Adrenal_Gland |
| ENSG00000206573 | 0.396928196 | 0.0484263 | Adrenal_Gland |
| ENSG00000168137 | 0.487554425 | 0.0511361 | Adrenal_Gland |
| ENSG00000134077 | 0.523330647 | -0.057922 | Adrenal_Gland |
| ENSG00000227110 | 0.659409162 | 0.1164 | Adrenal_Gland |
| ENSG00000070950 | 0.758361391 | -0.103899 | Adrenal_Gland |
| ENSG00000254485 | 0.862538278 | -0.169903 | Adrenal_Gland |
| ENSG00000071282 | 2.683369274 | 0.238544 | Adrenal_Gland |
| ENSG00000206573 | 0.010658324 | 0.000843635 | Artery_Aorta |
| ENSG00000180914 | 0.038529546 | -0.00604953 | Artery_Aorta |
| ENSG00000134077 | 0.052550593 | -0.00517021 | Artery_Aorta |
| ENSG00000196220 | 0.106131757 | 0.0162571 | Artery_Aorta |
| ENSG00000168137 | 0.107359532 | -0.00574356 | Artery_Aorta |
| ENSG00000125046 | 0.168721627 | 0.0319515 | Artery_Aorta |
| ENSG00000070950 | 0.225437782 | 0.0299719 | Artery_Aorta |
| ENSG00000071282 | 0.60866609 | -0.0460039 | Artery_Aorta |
| ENSG00000182533 | 0.769630281 | -0.11865 | Artery_Aorta |
| ENSG00000156959 | 0.834571665 | -0.108393 | Artery_Aorta |
| ENSG00000227110 | 1.746303462 | 0.13073 | Artery_Aorta |
| ENSG00000206573 | 0.012257785 | 0.00193981 | Artery_Coronary |
| ENSG00000227110 | 0.031522655 | 0.00672162 | Artery_Coronary |
| ENSG00000125046 | 0.16594189 | -0.0346733 | Artery_Coronary |
| ENSG00000182533 | 0.327113736 | -0.0466533 | Artery_Coronary |
| ENSG00000168137 | 0.348471951 | -0.0303822 | Artery_Coronary |
| ENSG00000071282 | 0.39371647 | 0.0622865 | Artery_Coronary |
| ENSG00000156959 | 0.415577176 | 0.0864104 | Artery_Coronary |
| ENSG00000070950 | 0.581501498 | -0.0844078 | Artery_Coronary |
| ENSG00000196220 | 0.581995523 | 0.0808634 | Artery_Coronary |
| ENSG00000180914 | 0.651749671 | 0.108041 | Artery_Coronary |
| ENSG00000134077 | 0.828472461 | 0.0698421 | Artery_Coronary |
| ENSG00000180914 | 0.123932918 | -0.014542 | Artery_Tibial |
| ENSG00000156959 | 0.134693441 | -0.0191625 | Artery_Tibial |
| ENSG00000196220 | 0.176870866 | -0.0216449 | Artery_Tibial |
| ENSG00000125046 | 0.315620588 | -0.0349422 | Artery_Tibial |
| ENSG00000168137 | 0.363705724 | 0.0135579 | Artery_Tibial |
| ENSG00000182533 | 0.392347712 | -0.0352749 | Artery_Tibial |
| ENSG00000134077 | 0.476548798 | -0.0295754 | Artery_Tibial |
| ENSG00000070950 | 0.766331381 | 0.0556535 | Artery_Tibial |
| ENSG00000071282 | 1.153487844 | 0.0589085 | Artery_Tibial |
| ENSG00000206573 | 1.318178282 | 0.0481146 | Artery_Tibial |
| ENSG00000227110 | 2.559435658 | 0.134 | Artery_Tibial |
| ENSG00000134077 | 0.192743768 | -0.0132897 | B-cell_naive |
| ENSG00000168137 | 0.423613725 | 0.0151696 | B-cell_naive |
| ENSG00000254485 | 0.50312153 | 0.181316 | B-cell_naive |
| ENSG00000070950 | 0.669233252 | -0.029138 | B-cell_naive |
| ENSG00000206573 | 0.762488936 | 0.048967 | B-cell_naive |
| ENSG00000070950 | 0.075729503 | -0.00313402 | blood |
| ENSG00000206573 | 0.3245718 | 0.0125136 | blood |
| ENSG00000168137 | 0.429168794 | 0.00717146 | blood |
| ENSG00000180914 | 0.558957188 | -0.0592865 | blood |
| ENSG00000134077 | 1.030692209 | 0.0215252 | blood |
| ENSG00000134077 | 0.10436257 | -0.00309615 | brain |
| ENSG00000156959 | 0.171114638 | -0.00802112 | brain |
| ENSG00000224808 | 0.186479724 | 0.0154696 | brain |
| ENSG00000168137 | 0.192826358 | 0.00621137 | brain |
| ENSG00000180914 | 0.269079708 | -0.0314676 | brain |
| ENSG00000206573 | 0.338497771 | 0.0104317 | brain |
| ENSG00000227110 | 0.552160042 | 0.0323206 | brain |
| ENSG00000196220 | 0.555527369 | 0.0146947 | brain |
| ENSG00000235830 | 0.659155488 | 0.0490681 | brain |
| ENSG00000254485 | 0.835998291 | -0.0400191 | brain |
| ENSG00000070950 | 0.982682792 | 0.0340751 | brain |
| ENSG00000224884 | 1.045883454 | 0.0664252 | brain |
| ENSG00000071282 | 1.368226576 | 0.058263 | brain |
| ENSG00000180914 | 0.030693607 | 0.0112218 | Brain_Amygdala |
| ENSG00000168137 | 0.074462567 | 0.0133924 | Brain_Amygdala |
| ENSG00000270207 | 0.113445545 | 0.0381856 | Brain_Amygdala |
| ENSG00000196220 | 0.115493507 | -0.0197942 | Brain_Amygdala |
| ENSG00000070950 | 0.149955088 | 0.0466248 | Brain_Amygdala |
| ENSG00000206573 | 0.236483659 | -0.0586497 | Brain_Amygdala |
| ENSG00000134077 | 0.321387853 | 0.06726 | Brain_Amygdala |
| ENSG00000071282 | 0.510031682 | 0.132079 | Brain_Amygdala |
| ENSG00000125046 | 0.629209435 | -0.136286 | Brain_Amygdala |
| ENSG00000156959 | 0.81562376 | -0.066977 | Brain_Amygdala |
| ENSG00000254485 | 1.467024073 | -0.289116 | Brain_Amygdala |
| ENSG00000235830 | 1.540426594 | 0.283327 | Brain_Amygdala |
| ENSG00000227110 | 1.843902338 | 0.226811 | Brain_Amygdala |
| ENSG00000168137 | 0.018814956 | 0.00371311 | Brain_Anterior_cingulate_cortex_BA24 |
| ENSG00000182533 | 0.032557802 | 0.0111278 | Brain_Anterior_cingulate_cortex_BA24 |
| ENSG00000206573 | 0.111524523 | -0.0324019 | Brain_Anterior_cingulate_cortex_BA24 |
| ENSG00000214041 | 0.120588697 | 0.0287059 | Brain_Anterior_cingulate_cortex_BA24 |
| ENSG00000134077 | 0.120783659 | -0.0269221 | Brain_Anterior_cingulate_cortex_BA24 |
| ENSG00000070950 | 0.162925697 | 0.0384222 | Brain_Anterior_cingulate_cortex_BA24 |
| ENSG00000270207 | 0.348278224 | 0.0902736 | Brain_Anterior_cingulate_cortex_BA24 |
| ENSG00000125046 | 0.351724904 | -0.0865818 | Brain_Anterior_cingulate_cortex_BA24 |
| ENSG00000196220 | 0.381462421 | 0.0369934 | Brain_Anterior_cingulate_cortex_BA24 |
| ENSG00000180914 | 0.522009579 | 0.0878817 | Brain_Anterior_cingulate_cortex_BA24 |
| ENSG00000071282 | 0.815149641 | 0.151264 | Brain_Anterior_cingulate_cortex_BA24 |
| ENSG00000227110 | 0.895792949 | 0.116067 | Brain_Anterior_cingulate_cortex_BA24 |
| ENSG00000254485 | 0.908020182 | 0.196508 | Brain_Anterior_cingulate_cortex_BA24 |
| ENSG00000156959 | 0.997920374 | -0.0531012 | Brain_Anterior_cingulate_cortex_BA24 |
| ENSG00000235830 | 1.223182632 | 0.21357 | Brain_Anterior_cingulate_cortex_BA24 |
| ENSG00000180914 | 0.00377737 | 0.000905149 | Brain_Caudate_basal_ganglia |
| ENSG00000270207 | 0.116279528 | 0.0317516 | Brain_Caudate_basal_ganglia |
| ENSG00000254485 | 0.127352 | -0.0355924 | Brain_Caudate_basal_ganglia |
| ENSG00000156959 | 0.140228443 | -0.00889703 | Brain_Caudate_basal_ganglia |
| ENSG00000070950 | 0.237847559 | 0.044082 | Brain_Caudate_basal_ganglia |
| ENSG00000125046 | 0.272574245 | 0.0518561 | Brain_Caudate_basal_ganglia |
| ENSG00000071282 | 0.399155561 | -0.0548681 | Brain_Caudate_basal_ganglia |
| ENSG00000168137 | 0.430107822 | -0.0491935 | Brain_Caudate_basal_ganglia |
| ENSG00000227110 | 0.556277139 | 0.0818068 | Brain_Caudate_basal_ganglia |
| ENSG00000182533 | 0.573594479 | -0.108847 | Brain_Caudate_basal_ganglia |
| ENSG00000235830 | 0.67997841 | 0.122496 | Brain_Caudate_basal_ganglia |
| ENSG00000206573 | 0.75634693 | -0.0818242 | Brain_Caudate_basal_ganglia |
| ENSG00000134077 | 0.789007683 | -0.0907631 | Brain_Caudate_basal_ganglia |
| ENSG00000196220 | 1.140626298 | 0.103965 | Brain_Caudate_basal_ganglia |
| ENSG00000227929 | 0.056696952 | 0.015924 | Brain_Cerebellar_Hemisphere |
| ENSG00000070950 | 0.06443631 | 0.0122743 | Brain_Cerebellar_Hemisphere |
| ENSG00000206573 | 0.077668543 | -0.0120912 | Brain_Cerebellar_Hemisphere |
| ENSG00000182533 | 0.110549822 | 0.0278222 | Brain_Cerebellar_Hemisphere |
| ENSG00000156959 | 0.12440228 | 0.0185914 | Brain_Cerebellar_Hemisphere |
| ENSG00000125046 | 0.136841965 | 0.0389525 | Brain_Cerebellar_Hemisphere |
| ENSG00000196220 | 0.144637752 | 0.0198366 | Brain_Cerebellar_Hemisphere |
| ENSG00000254485 | 0.161987574 | 0.0481804 | Brain_Cerebellar_Hemisphere |
| ENSG00000270207 | 0.19718866 | 0.0499342 | Brain_Cerebellar_Hemisphere |
| ENSG00000168137 | 0.365847312 | -0.0356762 | Brain_Cerebellar_Hemisphere |
| ENSG00000228723 | 0.427379293 | -0.0915617 | Brain_Cerebellar_Hemisphere |
| ENSG00000071282 | 0.516478316 | -0.0545909 | Brain_Cerebellar_Hemisphere |
| ENSG00000227110 | 0.520889895 | 0.078277 | Brain_Cerebellar_Hemisphere |
| ENSG00000134077 | 0.640895278 | -0.0738326 | Brain_Cerebellar_Hemisphere |
| ENSG00000180914 | 0.825722082 | -0.105744 | Brain_Cerebellar_Hemisphere |
| ENSG00000168137 | 0.038391464 | -0.00512387 | Brain_Cerebellum |
| ENSG00000206573 | 0.048412372 | -0.00811305 | Brain_Cerebellum |
| ENSG00000125046 | 0.069465095 | 0.0213209 | Brain_Cerebellum |
| ENSG00000180914 | 0.10638913 | -0.0202687 | Brain_Cerebellum |
| ENSG00000071282 | 0.183233223 | -0.0242589 | Brain_Cerebellum |
| ENSG00000227929 | 0.185921626 | 0.0501901 | Brain_Cerebellum |
| ENSG00000270207 | 0.278039427 | 0.0610447 | Brain_Cerebellum |
| ENSG00000196220 | 0.426710561 | 0.0536103 | Brain_Cerebellum |
| ENSG00000182533 | 0.468660335 | -0.0947259 | Brain_Cerebellum |
| ENSG00000254485 | 0.498032 | 0.113424 | Brain_Cerebellum |
| ENSG00000228723 | 0.590105745 | 0.122087 | Brain_Cerebellum |
| ENSG00000134077 | 0.676861388 | -0.0791909 | Brain_Cerebellum |
| ENSG00000070950 | 1.211285214 | 0.135335 | Brain_Cerebellum |
| ENSG00000227110 | 1.528114475 | 0.162641 | Brain_Cerebellum |
| ENSG00000156959 | 1.607955858 | -0.133394 | Brain_Cerebellum |
| ENSG00000206573 | 0.002369012 | 0.000424172 | Brain_Cortex |
| ENSG00000180914 | 0.02523324 | 0.00584303 | Brain_Cortex |
| ENSG00000125046 | 0.107438503 | 0.0274712 | Brain_Cortex |
| ENSG00000168137 | 0.129875374 | 0.0189839 | Brain_Cortex |
| ENSG00000182533 | 0.215464011 | 0.0561568 | Brain_Cortex |
| ENSG00000070950 | 0.333414634 | 0.050582 | Brain_Cortex |
| ENSG00000214041 | 0.354985687 | 0.0756651 | Brain_Cortex |
| ENSG00000156959 | 0.770093007 | 0.038399 | Brain_Cortex |
| ENSG00000235830 | 0.782187169 | 0.126956 | Brain_Cortex |
| ENSG00000134077 | 0.785869086 | -0.11237 | Brain_Cortex |
| ENSG00000196220 | 0.982499211 | 0.0551246 | Brain_Cortex |
| ENSG00000270207 | 1.022245309 | 0.163687 | Brain_Cortex |
| ENSG00000071282 | 1.206391191 | 0.121825 | Brain_Cortex |
| ENSG00000227110 | 2.387175893 | 0.233343 | Brain_Cortex |
| ENSG00000125046 | 0.015191949 | 0.00494705 | Brain_Frontal_Cortex_BA9 |
| ENSG00000156959 | 0.020222702 | 0.00192434 | Brain_Frontal_Cortex_BA9 |
| ENSG00000180914 | 0.025573977 | 0.00615074 | Brain_Frontal_Cortex_BA9 |
| ENSG00000270207 | 0.026241491 | 0.0087978 | Brain_Frontal_Cortex_BA9 |
| ENSG00000168137 | 0.081729504 | -0.0120827 | Brain_Frontal_Cortex_BA9 |
| ENSG00000196220 | 0.167873818 | 0.0145699 | Brain_Frontal_Cortex_BA9 |
| ENSG00000206573 | 0.210929319 | -0.0387051 | Brain_Frontal_Cortex_BA9 |
| ENSG00000214041 | 0.221830654 | 0.0536703 | Brain_Frontal_Cortex_BA9 |
| ENSG00000235830 | 0.250136969 | -0.0646709 | Brain_Frontal_Cortex_BA9 |
| ENSG00000182533 | 0.265110601 | 0.0681203 | Brain_Frontal_Cortex_BA9 |
| ENSG00000071282 | 0.382427271 | 0.0685405 | Brain_Frontal_Cortex_BA9 |
| ENSG00000227110 | 0.410076078 | 0.0797805 | Brain_Frontal_Cortex_BA9 |
| ENSG00000134077 | 0.414512198 | -0.0741782 | Brain_Frontal_Cortex_BA9 |
| ENSG00000070950 | 0.578688992 | -0.0860847 | Brain_Frontal_Cortex_BA9 |
| ENSG00000070950 | 0.074080733 | -0.020965 | Brain_Hippocampus |
| ENSG00000180914 | 0.082219402 | -0.0231931 | Brain_Hippocampus |
| ENSG00000071282 | 0.09502601 | -0.0206423 | Brain_Hippocampus |
| ENSG00000156959 | 0.15821833 | -0.0107616 | Brain_Hippocampus |
| ENSG00000134077 | 0.168179478 | 0.0357906 | Brain_Hippocampus |
| ENSG00000125046 | 0.272870461 | -0.067709 | Brain_Hippocampus |
| ENSG00000206573 | 0.337856279 | -0.0687612 | Brain_Hippocampus |
| ENSG00000235830 | 0.358473341 | 0.0971943 | Brain_Hippocampus |
| ENSG00000227110 | 0.394525795 | 0.0478927 | Brain_Hippocampus |
| ENSG00000196220 | 0.452461576 | -0.0386742 | Brain_Hippocampus |
| ENSG00000270207 | 0.523903441 | 0.103705 | Brain_Hippocampus |
| ENSG00000254485 | 0.74696019 | 0.167499 | Brain_Hippocampus |
| ENSG00000168137 | 0.828179971 | -0.0728589 | Brain_Hippocampus |
| ENSG00000214041 | 0.906974264 | -0.146266 | Brain_Hippocampus |
| ENSG00000227110 | 0.101179416 | 0.0193047 | Brain_Hypothalamus |
| ENSG00000180914 | 0.129584974 | -0.0301768 | Brain_Hypothalamus |
| ENSG00000125046 | 0.186571809 | -0.050645 | Brain_Hypothalamus |
| ENSG00000071282 | 0.277720732 | -0.0505353 | Brain_Hypothalamus |
| ENSG00000196220 | 0.313966442 | 0.0269553 | Brain_Hypothalamus |
| ENSG00000182533 | 0.378094965 | 0.102211 | Brain_Hypothalamus |
| ENSG00000270207 | 0.484097028 | 0.123619 | Brain_Hypothalamus |
| ENSG00000254485 | 0.497809211 | 0.126289 | Brain_Hypothalamus |
| ENSG00000206573 | 0.507447731 | 0.0930223 | Brain_Hypothalamus |
| ENSG00000156959 | 0.512060263 | -0.046166 | Brain_Hypothalamus |
| ENSG00000134077 | 0.99375223 | -0.14073 | Brain_Hypothalamus |
| ENSG00000168137 | 1.295472857 | 0.116615 | Brain_Hypothalamus |
| ENSG00000070950 | 1.480112967 | 0.209055 | Brain_Hypothalamus |
| ENSG00000235830 | 2.500497119 | 0.332892 | Brain_Hypothalamus |
| ENSG00000254485 | 0.017579504 | -0.00172162 | brain_naive |
| ENSG00000235830 | 0.035475913 | 0.00411892 | brain_naive |
| ENSG00000070950 | 0.041480074 | -0.00216497 | brain_naive |
| ENSG00000180914 | 0.065584881 | -0.0100472 | brain_naive |
| ENSG00000168137 | 0.16066276 | 0.00379736 | brain_naive |
| ENSG00000270207 | 0.209142255 | 0.015831 | brain_naive |
| ENSG00000156959 | 0.331169765 | 0.00857791 | brain_naive |
| ENSG00000196220 | 0.512317329 | -0.00935078 | brain_naive |
| ENSG00000134077 | 0.78498141 | -0.0160634 | brain_naive |
| ENSG00000206573 | 0.85236077 | -0.026066 | brain_naive |
| ENSG00000227110 | 1.105983172 | 0.0461031 | brain_naive |
| ENSG00000071282 | 1.190851882 | 0.0622056 | brain_naive |
| ENSG00000231401 | 1.531512359 | 0.0774016 | brain_naive |
| ENSG00000125046 | 2.098764605 | -0.0680761 | brain_naive |
| ENSG00000125046 | 0.011876001 | 0.0028494 | Brain_Nucleus_accumbens_basal_ganglia |
| ENSG00000206573 | 0.054187857 | 0.00921077 | Brain_Nucleus_accumbens_basal_ganglia |
| ENSG00000168137 | 0.13697649 | -0.0176573 | Brain_Nucleus_accumbens_basal_ganglia |
| ENSG00000235830 | 0.179502323 | 0.0458207 | Brain_Nucleus_accumbens_basal_ganglia |
| ENSG00000180914 | 0.18190217 | -0.0400442 | Brain_Nucleus_accumbens_basal_ganglia |
| ENSG00000270207 | 0.191181575 | 0.0513108 | Brain_Nucleus_accumbens_basal_ganglia |
| ENSG00000182533 | 0.308147899 | 0.0758306 | Brain_Nucleus_accumbens_basal_ganglia |
| ENSG00000156959 | 0.350299164 | 0.0270846 | Brain_Nucleus_accumbens_basal_ganglia |
| ENSG00000134077 | 0.423689754 | -0.0579458 | Brain_Nucleus_accumbens_basal_ganglia |
| ENSG00000254485 | 0.473748624 | 0.115506 | Brain_Nucleus_accumbens_basal_ganglia |
| ENSG00000071282 | 0.51768965 | -0.0640858 | Brain_Nucleus_accumbens_basal_ganglia |
| ENSG00000070950 | 0.992435705 | 0.132515 | Brain_Nucleus_accumbens_basal_ganglia |
| ENSG00000227110 | 1.148819342 | -0.107688 | Brain_Nucleus_accumbens_basal_ganglia |
| ENSG00000196220 | 1.948963305 | 0.158926 | Brain_Nucleus_accumbens_basal_ganglia |
| ENSG00000227110 | 0.001340998 | -0.00027785 | Brain_Putamen_basal_ganglia |
| ENSG00000071282 | 0.0033662 | -0.000767038 | Brain_Putamen_basal_ganglia |
| ENSG00000125046 | 0.020250913 | 0.00641777 | Brain_Putamen_basal_ganglia |
| ENSG00000156959 | 0.072045837 | 0.00570281 | Brain_Putamen_basal_ganglia |
| ENSG00000180914 | 0.086730946 | -0.0210669 | Brain_Putamen_basal_ganglia |
| ENSG00000134077 | 0.128578277 | 0.0222503 | Brain_Putamen_basal_ganglia |
| ENSG00000182533 | 0.130844582 | -0.035936 | Brain_Putamen_basal_ganglia |
| ENSG00000196220 | 0.142873842 | 0.0269029 | Brain_Putamen_basal_ganglia |
| ENSG00000206573 | 0.278782309 | -0.0469844 | Brain_Putamen_basal_ganglia |
| ENSG00000254485 | 0.321146697 | 0.0974047 | Brain_Putamen_basal_ganglia |
| ENSG00000168137 | 0.326552385 | -0.0327951 | Brain_Putamen_basal_ganglia |
| ENSG00000235830 | 0.481794124 | -0.112767 | Brain_Putamen_basal_ganglia |
| ENSG00000070950 | 0.747569338 | 0.136068 | Brain_Putamen_basal_ganglia |
| ENSG00000270207 | 1.000890347 | -0.211812 | Brain_Putamen_basal_ganglia |
| ENSG00000182533 | 0.141112861 | -0.0445792 | Brain_Spinal_cord_cervical_c-1 |
| ENSG00000254485 | 0.149411961 | 0.0587144 | Brain_Spinal_cord_cervical_c-1 |
| ENSG00000070950 | 0.150570758 | -0.038467 | Brain_Spinal_cord_cervical_c-1 |
| ENSG00000196220 | 0.151076592 | 0.0314919 | Brain_Spinal_cord_cervical_c-1 |
| ENSG00000270207 | 0.18557992 | -0.0508658 | Brain_Spinal_cord_cervical_c-1 |
| ENSG00000071282 | 0.220293146 | 0.0470104 | Brain_Spinal_cord_cervical_c-1 |
| ENSG00000180914 | 0.32788551 | -0.100164 | Brain_Spinal_cord_cervical_c-1 |
| ENSG00000125046 | 0.395793393 | -0.125612 | Brain_Spinal_cord_cervical_c-1 |
| ENSG00000206573 | 0.440275266 | 0.0916502 | Brain_Spinal_cord_cervical_c-1 |
| ENSG00000227110 | 0.470572212 | 0.0857166 | Brain_Spinal_cord_cervical_c-1 |
| ENSG00000134077 | 0.654197089 | 0.097509 | Brain_Spinal_cord_cervical_c-1 |
| ENSG00000168137 | 0.983041834 | 0.104281 | Brain_Spinal_cord_cervical_c-1 |
| ENSG00000156959 | 1.049091644 | -0.130806 | Brain_Spinal_cord_cervical_c-1 |
| ENSG00000235830 | 1.499715486 | 0.300069 | Brain_Spinal_cord_cervical_c-1 |
| ENSG00000070950 | 0.040412504 | 0.0134814 | Brain_Substantia_nigra |
| ENSG00000182533 | 0.147428521 | 0.0460683 | Brain_Substantia_nigra |
| ENSG00000235830 | 0.155321567 | -0.0536843 | Brain_Substantia_nigra |
| ENSG00000254485 | 0.185190583 | -0.0666817 | Brain_Substantia_nigra |
| ENSG00000180914 | 0.250126154 | -0.0501966 | Brain_Substantia_nigra |
| ENSG00000206573 | 0.254544196 | 0.0539146 | Brain_Substantia_nigra |
| ENSG00000168137 | 0.279327292 | 0.0423986 | Brain_Substantia_nigra |
| ENSG00000196220 | 0.368906446 | 0.0493348 | Brain_Substantia_nigra |
| ENSG00000156959 | 0.473517274 | -0.0484137 | Brain_Substantia_nigra |
| ENSG00000125046 | 0.560264783 | -0.15344 | Brain_Substantia_nigra |
| ENSG00000270207 | 0.660202851 | -0.148742 | Brain_Substantia_nigra |
| ENSG00000134077 | 0.746572334 | -0.120179 | Brain_Substantia_nigra |
| ENSG00000071282 | 0.839558482 | -0.169182 | Brain_Substantia_nigra |
| ENSG00000227110 | 1.504444562 | 0.161664 | Brain_Substantia_nigra |
| ENSG00000071282 | 0.037585201 | 0.0027293 | Breast_Mammary_Tissue |
| ENSG00000070950 | 0.081313996 | -0.00669477 | Breast_Mammary_Tissue |
| ENSG00000134077 | 0.1626275 | -0.0176633 | Breast_Mammary_Tissue |
| ENSG00000206573 | 0.35260627 | -0.0259041 | Breast_Mammary_Tissue |
| ENSG00000156959 | 0.434485111 | 0.0650856 | Breast_Mammary_Tissue |
| ENSG00000227110 | 0.457621541 | 0.0575125 | Breast_Mammary_Tissue |
| ENSG00000125046 | 0.599672733 | 0.0654899 | Breast_Mammary_Tissue |
| ENSG00000180914 | 0.664084977 | -0.0470028 | Breast_Mammary_Tissue |
| ENSG00000182533 | 0.92550172 | 0.10872 | Breast_Mammary_Tissue |
| ENSG00000168137 | 0.965090927 | 0.0407951 | Breast_Mammary_Tissue |
| ENSG00000254485 | 1.025938955 | 0.116652 | Breast_Mammary_Tissue |
| ENSG00000196220 | 1.535140997 | -0.0633363 | Breast_Mammary_Tissue |
| ENSG00000134077 | 0.011465568 | -0.000631654 | CD4_T-cell_anti-CD3-CD28 |
| ENSG00000206573 | 0.520139764 | 0.031484 | CD4_T-cell_anti-CD3-CD28 |
| ENSG00000070950 | 0.548720281 | 0.0231119 | CD4_T-cell_anti-CD3-CD28 |
| ENSG00000168137 | 0.586665024 | 0.0128332 | CD4_T-cell_anti-CD3-CD28 |
| ENSG00000180914 | 0.07402717 | 0.0322977 | CD4_T-cell_naive |
| ENSG00000196220 | 0.082544382 | 0.0163852 | CD4_T-cell_naive |
| ENSG00000134077 | 0.115007634 | -0.00718378 | CD4_T-cell_naive |
| ENSG00000070950 | 0.197484192 | -0.01196 | CD4_T-cell_naive |
| ENSG00000254485 | 0.227564599 | 0.101148 | CD4_T-cell_naive |
| ENSG00000206573 | 0.583020177 | 0.0442674 | CD4_T-cell_naive |
| ENSG00000168137 | 0.763437548 | -0.0213432 | CD4_T-cell_naive |
| ENSG00000168137 | 0.093110859 | 0.00314705 | CD8_T-cell_anti-CD3-CD28 |
| ENSG00000206573 | 0.322394872 | 0.0236154 | CD8_T-cell_anti-CD3-CD28 |
| ENSG00000070950 | 0.959484686 | 0.0334977 | CD8_T-cell_anti-CD3-CD28 |
| ENSG00000134077 | 1.864298207 | -0.0425541 | CD8_T-cell_anti-CD3-CD28 |
| ENSG00000235830 | 0.099631232 | 0.0258906 | CD8_T-cell_naive |
| ENSG00000196220 | 0.158303988 | -0.0372807 | CD8_T-cell_naive |
| ENSG00000134077 | 0.512648066 | -0.0222658 | CD8_T-cell_naive |
| ENSG00000168137 | 0.561353243 | -0.0136999 | CD8_T-cell_naive |
| ENSG00000206573 | 0.609305057 | 0.0377657 | CD8_T-cell_naive |
| ENSG00000070950 | 0.807443697 | -0.0323022 | CD8_T-cell_naive |
| ENSG00000254485 | 0.885482422 | 0.280731 | CD8_T-cell_naive |
| ENSG00000180914 | 0.965752671 | 0.226987 | CD8_T-cell_naive |
| ENSG00000156959 | 0.060232721 | 0.00951884 | Cells_Cultured_fibroblasts |
| ENSG00000168137 | 0.079696103 | -0.00492391 | Cells_Cultured_fibroblasts |
| ENSG00000227110 | 0.120755556 | 0.0183656 | Cells_Cultured_fibroblasts |
| ENSG00000206573 | 0.180841837 | 0.0140931 | Cells_Cultured_fibroblasts |
| ENSG00000254485 | 0.248427615 | -0.0399495 | Cells_Cultured_fibroblasts |
| ENSG00000070950 | 0.265795634 | 0.0114226 | Cells_Cultured_fibroblasts |
| ENSG00000196220 | 0.431404069 | 0.037292 | Cells_Cultured_fibroblasts |
| ENSG00000134077 | 0.585753869 | -0.0335963 | Cells_Cultured_fibroblasts |
| ENSG00000071282 | 0.972083936 | 0.0309447 | Cells_Cultured_fibroblasts |
| ENSG00000180914 | 1.028994277 | -0.065577 | Cells_Cultured_fibroblasts |
| ENSG00000168137 | 0.110871489 | 0.0137191 | Cells_EBV-transformed_lymphocytes |
| ENSG00000071282 | 0.116414076 | 0.0357012 | Cells_EBV-transformed_lymphocytes |
| ENSG00000196220 | 0.14582214 | 0.0437702 | Cells_EBV-transformed_lymphocytes |
| ENSG00000254485 | 0.302063107 | -0.0943479 | Cells_EBV-transformed_lymphocytes |
| ENSG00000182533 | 0.321008434 | -0.0847512 | Cells_EBV-transformed_lymphocytes |
| ENSG00000180914 | 0.493024399 | -0.0674334 | Cells_EBV-transformed_lymphocytes |
| ENSG00000070950 | 0.495245138 | 0.0762336 | Cells_EBV-transformed_lymphocytes |
| ENSG00000134077 | 0.603596635 | -0.112857 | Cells_EBV-transformed_lymphocytes |
| ENSG00000206573 | 1.165770733 | 0.152024 | Cells_EBV-transformed_lymphocytes |
| ENSG00000125046 | 1.393762707 | -0.238672 | Cells_EBV-transformed_lymphocytes |
| ENSG00000227110 | 0.014598228 | 0.00251665 | Colon_Sigmoid |
| ENSG00000206573 | 0.083151418 | -0.00848826 | Colon_Sigmoid |
| ENSG00000180914 | 0.102465825 | 0.0184555 | Colon_Sigmoid |
| ENSG00000168137 | 0.147922769 | -0.0111641 | Colon_Sigmoid |
| ENSG00000196220 | 0.148646239 | 0.0180577 | Colon_Sigmoid |
| ENSG00000125046 | 0.306084956 | -0.048117 | Colon_Sigmoid |
| ENSG00000254485 | 0.449440242 | 0.0741896 | Colon_Sigmoid |
| ENSG00000071282 | 0.610201547 | 0.0579038 | Colon_Sigmoid |
| ENSG00000070950 | 1.25796888 | 0.101062 | Colon_Sigmoid |
| ENSG00000134077 | 1.324969949 | -0.0784203 | Colon_Sigmoid |
| ENSG00000182533 | 1.7345053 | 0.183197 | Colon_Sigmoid |
| ENSG00000156959 | 1.918454676 | 0.119079 | Colon_Sigmoid |
| ENSG00000156959 | 0.051933434 | -0.00837208 | Colon_Transverse |
| ENSG00000227110 | 0.127103435 | 0.0176781 | Colon_Transverse |
| ENSG00000125046 | 0.390005627 | -0.0413981 | Colon_Transverse |
| ENSG00000070950 | 0.456028752 | 0.0335791 | Colon_Transverse |
| ENSG00000182533 | 0.531314524 | 0.0665733 | Colon_Transverse |
| ENSG00000180914 | 0.663118185 | 0.0859058 | Colon_Transverse |
| ENSG00000206573 | 0.674876528 | 0.0433047 | Colon_Transverse |
| ENSG00000168137 | 0.91676621 | 0.0441469 | Colon_Transverse |
| ENSG00000196220 | 1.014265542 | 0.092052 | Colon_Transverse |
| ENSG00000254485 | 1.107996163 | 0.118237 | Colon_Transverse |
| ENSG00000134077 | 2.247734075 | -0.105878 | Colon_Transverse |
| ENSG00000071282 | 2.920594426 | 0.132248 | Colon_Transverse |
| ENSG00000156959 | 0.008846165 | -0.00136963 | Esophagus_Gastroesophageal_Junction |
| ENSG00000134077 | 0.035265782 | -0.00394507 | Esophagus_Gastroesophageal_Junction |
| ENSG00000227110 | 0.036460547 | -0.00724339 | Esophagus_Gastroesophageal_Junction |
| ENSG00000196220 | 0.09743311 | -0.0175614 | Esophagus_Gastroesophageal_Junction |
| ENSG00000182533 | 0.098280744 | -0.0183754 | Esophagus_Gastroesophageal_Junction |
| ENSG00000206573 | 0.119596896 | 0.0104493 | Esophagus_Gastroesophageal_Junction |
| ENSG00000070950 | 0.164085682 | 0.0254442 | Esophagus_Gastroesophageal_Junction |
| ENSG00000125046 | 0.463610763 | 0.0549207 | Esophagus_Gastroesophageal_Junction |
| ENSG00000254485 | 0.478861916 | 0.0911458 | Esophagus_Gastroesophageal_Junction |
| ENSG00000168137 | 0.765401737 | 0.0438042 | Esophagus_Gastroesophageal_Junction |
| ENSG00000180914 | 0.977035793 | -0.134016 | Esophagus_Gastroesophageal_Junction |
| ENSG00000071282 | 1.344975919 | 0.106289 | Esophagus_Gastroesophageal_Junction |
| ENSG00000168137 | 0.002078723 | 0.000180543 | Esophagus_Mucosa |
| ENSG00000196220 | 0.051758732 | -0.00520651 | Esophagus_Mucosa |
| ENSG00000134077 | 0.11130898 | 0.00805112 | Esophagus_Mucosa |
| ENSG00000156959 | 0.130426261 | 0.0217846 | Esophagus_Mucosa |
| ENSG00000070950 | 0.169732841 | 0.0130609 | Esophagus_Mucosa |
| ENSG00000227110 | 0.171108198 | -0.0275536 | Esophagus_Mucosa |
| ENSG00000206573 | 0.217321027 | -0.0177476 | Esophagus_Mucosa |
| ENSG00000125046 | 0.504576394 | 0.0570708 | Esophagus_Mucosa |
| ENSG00000182533 | 0.832059737 | -0.0956891 | Esophagus_Mucosa |
| ENSG00000180914 | 1.236083326 | -0.105189 | Esophagus_Mucosa |
| ENSG00000071282 | 2.263031721 | 0.128015 | Esophagus_Mucosa |
| ENSG00000254485 | 0.054786059 | 0.011124 | Esophagus_Muscularis |
| ENSG00000196220 | 0.186786082 | 0.0222055 | Esophagus_Muscularis |
| ENSG00000125046 | 0.201914512 | 0.022616 | Esophagus_Muscularis |
| ENSG00000168137 | 0.219073806 | 0.012767 | Esophagus_Muscularis |
| ENSG00000180914 | 0.306837825 | -0.0454131 | Esophagus_Muscularis |
| ENSG00000156959 | 0.399863845 | 0.0362616 | Esophagus_Muscularis |
| ENSG00000070950 | 0.401246689 | 0.0429161 | Esophagus_Muscularis |
| ENSG00000134077 | 0.679822546 | -0.0373572 | Esophagus_Muscularis |
| ENSG00000182533 | 0.687298082 | 0.0819048 | Esophagus_Muscularis |
| ENSG00000071282 | 0.712978013 | 0.048386 | Esophagus_Muscularis |
| ENSG00000206573 | 0.981170563 | 0.0411257 | Esophagus_Muscularis |
| ENSG00000227110 | 1.821448928 | 0.142784 | Esophagus_Muscularis |
| ENSG00000134077 | 0.080994122 | -0.00259314 | fat |
| ENSG00000196220 | 0.138478732 | -0.0128461 | fat |
| ENSG00000254485 | 0.208666616 | 0.0287278 | fat |
| ENSG00000206573 | 0.209908446 | -0.00871565 | fat |
| ENSG00000071282 | 0.297697875 | 0.0192986 | fat |
| ENSG00000168137 | 0.527421984 | 0.0111146 | fat |
| ENSG00000070950 | 0.554824076 | 0.0214381 | fat |
| ENSG00000125046 | 0.59334232 | -0.0710936 | fat |
| ENSG00000235830 | 0.633831156 | 0.0345025 | fat |
| ENSG00000206573 | 0.027802255 | 0.00332385 | fibroblast |
| ENSG00000071282 | 0.127168036 | 0.0257839 | fibroblast |
| ENSG00000168137 | 0.134804199 | 0.00674522 | fibroblast |
| ENSG00000070950 | 0.254727634 | -0.0194152 | fibroblast |
| ENSG00000180914 | 0.404443158 | -0.107797 | fibroblast |
| ENSG00000134077 | 0.777790935 | 0.0272546 | fibroblast |
| ENSG00000125046 | 0.013222893 | 0.00217858 | Heart_Atrial_Appendage |
| ENSG00000180914 | 0.145836114 | 0.0259611 | Heart_Atrial_Appendage |
| ENSG00000206573 | 0.165859166 | 0.0161321 | Heart_Atrial_Appendage |
| ENSG00000196220 | 0.415286674 | 0.0595311 | Heart_Atrial_Appendage |
| ENSG00000134077 | 0.557888832 | -0.0315302 | Heart_Atrial_Appendage |
| ENSG00000070950 | 0.584582562 | 0.0514645 | Heart_Atrial_Appendage |
| ENSG00000182533 | 0.614061431 | -0.0450076 | Heart_Atrial_Appendage |
| ENSG00000235830 | 0.865644365 | 0.101738 | Heart_Atrial_Appendage |
| ENSG00000254485 | 0.88813621 | 0.123547 | Heart_Atrial_Appendage |
| ENSG00000168137 | 1.004052577 | -0.0424248 | Heart_Atrial_Appendage |
| ENSG00000227110 | 1.537846174 | 0.132065 | Heart_Atrial_Appendage |
| ENSG00000071282 | 3.260029932 | 0.187137 | Heart_Atrial_Appendage |
| ENSG00000180914 | 0.014699296 | -0.00315069 | Heart_Left_Ventricle |
| ENSG00000235830 | 0.023400156 | -0.00343674 | Heart_Left_Ventricle |
| ENSG00000125046 | 0.057702679 | 0.0074496 | Heart_Left_Ventricle |
| ENSG00000254485 | 0.083931033 | -0.0168573 | Heart_Left_Ventricle |
| ENSG00000134077 | 0.141043147 | 0.00971668 | Heart_Left_Ventricle |
| ENSG00000196220 | 0.199838514 | 0.0180949 | Heart_Left_Ventricle |
| ENSG00000168137 | 0.218738782 | 0.0156406 | Heart_Left_Ventricle |
| ENSG00000206573 | 0.401302488 | 0.0377211 | Heart_Left_Ventricle |
| ENSG00000070950 | 0.538177449 | 0.0494228 | Heart_Left_Ventricle |
| ENSG00000182533 | 0.558358271 | -0.0405184 | Heart_Left_Ventricle |
| ENSG00000071282 | 0.5637276 | 0.0489793 | Heart_Left_Ventricle |
| ENSG00000254485 | 0.026023791 | -0.00668833 | iPSC |
| ENSG00000206573 | 0.057562828 | 0.00496148 | iPSC |
| ENSG00000156959 | 0.150610687 | 0.0158903 | iPSC |
| ENSG00000070950 | 0.428831753 | 0.0153007 | iPSC |
| ENSG00000071282 | 0.498899639 | 0.046555 | iPSC |
| ENSG00000180914 | 0.512430374 | -0.058474 | iPSC |
| ENSG00000134077 | 0.711299271 | -0.0260918 | iPSC |
| ENSG00000196220 | 0.838889656 | -0.0501338 | iPSC |
| ENSG00000168137 | 0.988349217 | -0.030439 | iPSC |
| ENSG00000227110 | 1.193473407 | 0.101234 | iPSC |
| ENSG00000224884 | 2.125325721 | 0.259687 | iPSC |
| ENSG00000180914 | 0.091624909 | -0.0403529 | Kidney_Cortex |
| ENSG00000206573 | 0.209010822 | -0.0587699 | Kidney_Cortex |
| ENSG00000196220 | 0.213383429 | -0.0567306 | Kidney_Cortex |
| ENSG00000254485 | 0.355965364 | 0.151102 | Kidney_Cortex |
| ENSG00000071282 | 0.496827844 | 0.117378 | Kidney_Cortex |
| ENSG00000125046 | 0.532560614 | 0.157052 | Kidney_Cortex |
| ENSG00000134077 | 0.828574879 | -0.194403 | Kidney_Cortex |
| ENSG00000070950 | 0.976315519 | 0.247115 | Kidney_Cortex |
| ENSG00000168137 | 1.017466912 | -0.132291 | Kidney_Cortex |
| ENSG00000227110 | 1.177921536 | 0.308731 | Kidney_Cortex |
| ENSG00000168137 | 0.0040517 | 0.00017861 | LCL |
| ENSG00000182533 | 0.024832985 | -0.00495068 | LCL |
| ENSG00000134077 | 0.051844361 | 0.00245263 | LCL |
| ENSG00000254485 | 0.071936141 | 0.0215732 | LCL |
| ENSG00000235830 | 0.187179525 | -0.0151685 | LCL |
| ENSG00000070950 | 0.34264388 | -0.0158507 | LCL |
| ENSG00000180914 | 0.439220877 | -0.0590668 | LCL |
| ENSG00000206573 | 0.567393039 | -0.0344203 | LCL |
| ENSG00000196220 | 1.394807044 | -0.132078 | LCL |
| ENSG00000071282 | 1.790949017 | 0.348039 | LCL |
| ENSG00000125046 | 0.015078625 | 0.00490173 | Liver |
| ENSG00000196220 | 0.250273731 | 0.0463005 | Liver |
| ENSG00000254485 | 0.307958991 | 0.0691243 | Liver |
| ENSG00000071282 | 0.474416225 | 0.0789179 | Liver |
| ENSG00000206573 | 0.481462372 | -0.0597322 | Liver |
| ENSG00000168137 | 0.497037853 | -0.058643 | Liver |
| ENSG00000134077 | 0.576013998 | -0.0587839 | Liver |
| ENSG00000070950 | 0.796026616 | 0.0963806 | Liver |
| ENSG00000180914 | 1.022960375 | -0.173067 | Liver |
| ENSG00000254485 | 0.089738548 | -0.0148219 | Lung |
| ENSG00000168137 | 0.175309365 | -0.00945692 | Lung |
| ENSG00000196220 | 0.262679315 | 0.0194544 | Lung |
| ENSG00000125046 | 0.315060432 | -0.0387519 | Lung |
| ENSG00000180914 | 0.476884629 | 0.0565444 | Lung |
| ENSG00000228723 | 0.496454443 | -0.0220288 | Lung |
| ENSG00000156959 | 0.83342189 | 0.0915636 | Lung |
| ENSG00000070950 | 0.84749762 | -0.0575317 | Lung |
| ENSG00000206573 | 1.141816638 | 0.0637588 | Lung |
| ENSG00000134077 | 1.170845521 | -0.0424453 | Lung |
| ENSG00000071282 | 1.217659978 | 0.0660317 | Lung |
| ENSG00000182533 | 1.227916047 | 0.0934752 | Lung |
| ENSG00000227110 | 1.443450263 | 0.10097 | Lung |
| ENSG00000180914 | 0.004039426 | -0.00229967 | macrophage_IFNg |
| ENSG00000206573 | 0.006174622 | -0.00110957 | macrophage_IFNg |
| ENSG00000125046 | 0.036923204 | -0.0437243 | macrophage_IFNg |
| ENSG00000134077 | 0.052910026 | -0.0239506 | macrophage_IFNg |
| ENSG00000168137 | 0.061590864 | 0.00408208 | macrophage_IFNg |
| ENSG00000070950 | 0.113508711 | 0.0115581 | macrophage_IFNg |
| ENSG00000071282 | 0.631571783 | -0.180583 | macrophage_IFNg |
| ENSG00000196220 | 0.270107405 | -0.0349452 | macrophage_Listeria |
| ENSG00000071282 | 0.304985266 | 0.0955919 | macrophage_Listeria |
| ENSG00000180914 | 0.473735697 | 0.126497 | macrophage_Listeria |
| ENSG00000134077 | 0.578453654 | -0.0293796 | macrophage_Listeria |
| ENSG00000168137 | 0.700401189 | -0.0310009 | macrophage_Listeria |
| ENSG00000206573 | 1.426275742 | -0.10323 | macrophage_Listeria |
| ENSG00000070950 | 2.039995638 | -0.102427 | macrophage_Listeria |
| ENSG00000134077 | 0.032480104 | 0.01466 | macrophage_naive |
| ENSG00000196220 | 0.054004377 | -0.0233046 | macrophage_naive |
| ENSG00000070950 | 0.1128403 | 0.0100579 | macrophage_naive |
| ENSG00000206573 | 0.137451826 | -0.0204684 | macrophage_naive |
| ENSG00000180914 | 0.162518255 | -0.0736727 | macrophage_naive |
| ENSG00000071282 | 0.281710943 | -0.0766251 | macrophage_naive |
| ENSG00000168137 | 0.759490689 | -0.0264047 | macrophage_naive |
| ENSG00000196220 | 0.024355462 | 0.00326379 | macrophage_Salmonella |
| ENSG00000070950 | 0.042473163 | 0.00507333 | macrophage_Salmonella |
| ENSG00000134077 | 0.044639898 | -0.0194758 | macrophage_Salmonella |
| ENSG00000180914 | 0.327512376 | 0.0959366 | macrophage_Salmonella |
| ENSG00000071282 | 0.410005743 | -0.130688 | macrophage_Salmonella |
| ENSG00000206573 | 0.655106906 | 0.0754091 | macrophage_Salmonella |
| ENSG00000168137 | 1.672536847 | 0.0567386 | macrophage_Salmonella |
| ENSG00000182533 | 0.083208223 | -0.0201833 | Minor_Salivary_Gland |
| ENSG00000156959 | 0.114025659 | -0.0386108 | Minor_Salivary_Gland |
| ENSG00000231401 | 0.125086422 | -0.0316573 | Minor_Salivary_Gland |
| ENSG00000071282 | 0.187095329 | -0.0319091 | Minor_Salivary_Gland |
| ENSG00000235830 | 0.339276781 | 0.0716908 | Minor_Salivary_Gland |
| ENSG00000196220 | 0.416548434 | 0.0568953 | Minor_Salivary_Gland |
| ENSG00000254485 | 0.82329248 | 0.18705 | Minor_Salivary_Gland |
| ENSG00000180914 | 0.885452397 | 0.18432 | Minor_Salivary_Gland |
| ENSG00000125046 | 0.909040446 | 0.157472 | Minor_Salivary_Gland |
| ENSG00000206573 | 0.996349936 | -0.0860147 | Minor_Salivary_Gland |
| ENSG00000168137 | 1.056680622 | 0.0850517 | Minor_Salivary_Gland |
| ENSG00000070950 | 1.737731685 | 0.267536 | Minor_Salivary_Gland |
| ENSG00000134077 | 3.107034342 | -0.309116 | Minor_Salivary_Gland |
| ENSG00000227110 | 3.406012096 | 0.352503 | Minor_Salivary_Gland |
| ENSG00000134077 | 0.105211124 | -0.00804651 | monocyte |
| ENSG00000168137 | 0.197093613 | -0.0070061 | monocyte |
| ENSG00000070950 | 0.245134336 | -0.0138818 | monocyte |
| ENSG00000206573 | 0.246556367 | 0.023737 | monocyte |
| ENSG00000196220 | 0.246612303 | -0.0283496 | monocyte |
| ENSG00000270207 | 0.474145697 | 0.0518654 | monocyte |
| ENSG00000134077 | 0.0989871 | -0.00559 | monocyte_CD16_naive |
| ENSG00000206573 | 0.672022254 | 0.0464757 | monocyte_CD16_naive |
| ENSG00000070950 | 0.708573425 | -0.0310182 | monocyte_CD16_naive |
| ENSG00000168137 | 0.776293842 | -0.0208096 | monocyte_CD16_naive |
| ENSG00000070950 | 0.142996365 | 0.0141342 | monocyte_IAV |
| ENSG00000206573 | 0.195842842 | -0.0183241 | monocyte_IAV |
| ENSG00000134077 | 0.233958395 | -0.0224452 | monocyte_IAV |
| ENSG00000156959 | 0.40208224 | -0.0607341 | monocyte_IAV |
| ENSG00000254485 | 0.588848507 | -0.101923 | monocyte_IAV |
| ENSG00000168137 | 0.960895352 | -0.0399812 | monocyte_IAV |
| ENSG00000206573 | 0.292400872 | -0.0282584 | monocyte_LPS |
| ENSG00000070950 | 0.312768994 | -0.0301145 | monocyte_LPS |
| ENSG00000134077 | 0.564663776 | -0.0342398 | monocyte_LPS |
| ENSG00000196220 | 0.841140164 | -0.118197 | monocyte_LPS |
| ENSG00000168137 | 0.996690291 | -0.0378476 | monocyte_LPS |
| ENSG00000070950 | 0.100253892 | 0.00687466 | monocyte_naive |
| ENSG00000134077 | 0.149753941 | -0.010904 | monocyte_naive |
| ENSG00000196220 | 0.302761064 | -0.0333456 | monocyte_naive |
| ENSG00000270207 | 0.394390085 | -0.115579 | monocyte_naive |
| ENSG00000206573 | 0.587290875 | -0.0462433 | monocyte_naive |
| ENSG00000168137 | 0.641956593 | -0.0312706 | monocyte_naive |
| ENSG00000196220 | 0.078311964 | -0.0142874 | monocyte_Pam3CSK4 |
| ENSG00000070950 | 0.217287362 | -0.0204145 | monocyte_Pam3CSK4 |
| ENSG00000134077 | 0.36581807 | -0.0247532 | monocyte_Pam3CSK4 |
| ENSG00000206573 | 0.373319928 | -0.0341207 | monocyte_Pam3CSK4 |
| ENSG00000168137 | 0.486448148 | -0.0227216 | monocyte_Pam3CSK4 |
| ENSG00000070950 | 0.2375165 | 0.0281008 | monocyte_R848 |
| ENSG00000196220 | 0.460552525 | -0.0755785 | monocyte_R848 |
| ENSG00000206573 | 0.863691733 | -0.0586743 | monocyte_R848 |
| ENSG00000134077 | 1.314101585 | -0.0598163 | monocyte_R848 |
| ENSG00000168137 | 1.823402359 | -0.0572188 | monocyte_R848 |
| ENSG00000134077 | 0.170876416 | 0.00471422 | muscle_naive |
| ENSG00000235830 | 0.2678162 | -0.0817928 | muscle_naive |
| ENSG00000196220 | 0.368976523 | -0.0292845 | muscle_naive |
| ENSG00000182533 | 0.426247929 | 0.0202164 | muscle_naive |
| ENSG00000070950 | 0.58610705 | 0.0225168 | muscle_naive |
| ENSG00000206573 | 0.83454496 | 0.0261753 | muscle_naive |
| ENSG00000168137 | 0.851246232 | 0.0185788 | muscle_naive |
| ENSG00000071282 | 1.831484839 | -0.0851582 | muscle_naive |
| ENSG00000224884 | 2.095086552 | 0.312903 | muscle_naive |
| ENSG00000134077 | 0.139007152 | 0.00852339 | Muscle_Skeletal |
| ENSG00000196220 | 0.170776016 | 0.0134872 | Muscle_Skeletal |
| ENSG00000206573 | 0.292680254 | -0.0179894 | Muscle_Skeletal |
| ENSG00000180914 | 0.297335146 | -0.0281436 | Muscle_Skeletal |
| ENSG00000125046 | 0.322608423 | 0.0376392 | Muscle_Skeletal |
| ENSG00000168137 | 0.397619835 | -0.0210555 | Muscle_Skeletal |
| ENSG00000254485 | 0.541759618 | -0.0567063 | Muscle_Skeletal |
| ENSG00000070950 | 0.748103956 | 0.0550601 | Muscle_Skeletal |
| ENSG00000182533 | 1.059905065 | -0.0470041 | Muscle_Skeletal |
| ENSG00000071282 | 1.746868043 | -0.052124 | Muscle_Skeletal |
| ENSG00000125046 | 0.10647958 | 0.0173869 | Nerve_Tibial |
| ENSG00000270207 | 0.179517424 | 0.0284111 | Nerve_Tibial |
| ENSG00000182533 | 0.221580294 | -0.0304516 | Nerve_Tibial |
| ENSG00000168137 | 0.22427162 | 0.0111157 | Nerve_Tibial |
| ENSG00000180914 | 0.294371452 | 0.0388972 | Nerve_Tibial |
| ENSG00000156959 | 0.427063375 | 0.0557576 | Nerve_Tibial |
| ENSG00000134077 | 0.439929499 | -0.0311625 | Nerve_Tibial |
| ENSG00000196220 | 0.816661814 | 0.0481804 | Nerve_Tibial |
| ENSG00000070950 | 0.880358169 | 0.0708467 | Nerve_Tibial |
| ENSG00000254485 | 1.055703917 | 0.117294 | Nerve_Tibial |
| ENSG00000206573 | 1.273974911 | 0.0507547 | Nerve_Tibial |
| ENSG00000227110 | 1.334710825 | 0.0810932 | Nerve_Tibial |
| ENSG00000071282 | 1.518814011 | 0.0771068 | Nerve_Tibial |
| ENSG00000134077 | 0.002018943 | 0.000237114 | neutrophil |
| ENSG00000206573 | 0.223855476 | 0.0119467 | neutrophil |
| ENSG00000070950 | 0.263278496 | 0.0145891 | neutrophil |
| ENSG00000168137 | 0.725325002 | -0.0183228 | neutrophil |
| ENSG00000168137 | 0.097534217 | -0.00506902 | NK-cell_naive |
| ENSG00000070950 | 0.1259318 | 0.00812756 | NK-cell_naive |
| ENSG00000206573 | 0.559853175 | 0.0442239 | NK-cell_naive |
| ENSG00000180914 | 0.585345809 | -0.200493 | NK-cell_naive |
| ENSG00000134077 | 0.759650473 | 0.0360657 | NK-cell_naive |
| ENSG00000156959 | 0.079743065 | 0.0223444 | Ovary |
| ENSG00000206573 | 0.123258088 | -0.0221574 | Ovary |
| ENSG00000134077 | 0.123602011 | -0.0186714 | Ovary |
| ENSG00000196220 | 0.215502529 | 0.0364599 | Ovary |
| ENSG00000125046 | 0.380305754 | -0.0934505 | Ovary |
| ENSG00000227110 | 0.588651392 | 0.0864861 | Ovary |
| ENSG00000168137 | 0.670349957 | -0.074659 | Ovary |
| ENSG00000070950 | 0.680078192 | -0.106567 | Ovary |
| ENSG00000180914 | 0.68966574 | -0.147797 | Ovary |
| ENSG00000182533 | 0.914616649 | 0.153916 | Ovary |
| ENSG00000071282 | 1.554682308 | 0.17745 | Ovary |
| ENSG00000206573 | 0.024845861 | 0.00401381 | Pancreas |
| ENSG00000168137 | 0.071541671 | -0.00752468 | Pancreas |
| ENSG00000070950 | 0.122413864 | -0.0244541 | Pancreas |
| ENSG00000180914 | 0.212557238 | -0.0391936 | Pancreas |
| ENSG00000254485 | 0.322010927 | -0.0678654 | Pancreas |
| ENSG00000156959 | 0.363030935 | -0.0397154 | Pancreas |
| ENSG00000196220 | 0.596733594 | 0.0677919 | Pancreas |
| ENSG00000134077 | 1.508387608 | 0.123395 | Pancreas |
| ENSG00000071282 | 1.774339289 | 0.163067 | Pancreas |
| ENSG00000125046 | 1.878988705 | 0.215544 | Pancreas |
| ENSG00000071282 | 0.011382636 | -0.00274093 | pancreatic_islet |
| ENSG00000224808 | 0.029722412 | -0.00759002 | pancreatic_islet |
| ENSG00000168137 | 0.041983989 | -0.00360716 | pancreatic_islet |
| ENSG00000235830 | 0.112007074 | -0.0370659 | pancreatic_islet |
| ENSG00000231401 | 0.142525175 | -0.043239 | pancreatic_islet |
| ENSG00000227929 | 0.206225675 | 0.0487547 | pancreatic_islet |
| ENSG00000228723 | 0.230119981 | 0.0849079 | pancreatic_islet |
| ENSG00000180914 | 0.239812823 | 0.0677809 | pancreatic_islet |
| ENSG00000206573 | 0.268739295 | 0.0311607 | pancreatic_islet |
| ENSG00000134077 | 0.268944157 | -0.0170086 | pancreatic_islet |
| ENSG00000125046 | 0.365514669 | -0.0851439 | pancreatic_islet |
| ENSG00000196220 | 0.367916423 | 0.0650276 | pancreatic_islet |
| ENSG00000224884 | 0.709182138 | 0.206464 | pancreatic_islet |
| ENSG00000254485 | 0.753523578 | 0.121516 | pancreatic_islet |
| ENSG00000070950 | 0.894534098 | -0.111684 | pancreatic_islet |
| ENSG00000156959 | 0.930209844 | -0.111691 | pancreatic_islet |
| ENSG00000227110 | 1.495836447 | -0.19912 | pancreatic_islet |
| ENSG00000228723 | 0.008735371 | -0.00199552 | Pituitary |
| ENSG00000071282 | 0.019570728 | -0.00349867 | Pituitary |
| ENSG00000196220 | 0.087777412 | -0.0138834 | Pituitary |
| ENSG00000180914 | 0.166221357 | -0.0301521 | Pituitary |
| ENSG00000070950 | 0.20520882 | 0.0304799 | Pituitary |
| ENSG00000156959 | 0.269242749 | -0.0270962 | Pituitary |
| ENSG00000235830 | 0.326076404 | -0.0723281 | Pituitary |
| ENSG00000254485 | 0.436249641 | 0.0999941 | Pituitary |
| ENSG00000168137 | 0.702195733 | -0.057238 | Pituitary |
| ENSG00000206573 | 0.721020168 | -0.0881608 | Pituitary |
| ENSG00000134077 | 0.95349313 | -0.107696 | Pituitary |
| ENSG00000125046 | 1.7815459 | 0.189693 | Pituitary |
| ENSG00000227110 | 5.353974853 | 0.460362 | Pituitary |
| ENSG00000182533 | 0.088272587 | -0.0189224 | Prostate |
| ENSG00000206573 | 0.110800859 | 0.0151944 | Prostate |
| ENSG00000070950 | 0.251957021 | 0.039203 | Prostate |
| ENSG00000180914 | 0.352542698 | 0.0718804 | Prostate |
| ENSG00000254485 | 0.356352922 | 0.0680345 | Prostate |
| ENSG00000134077 | 0.360220759 | -0.0422244 | Prostate |
| ENSG00000168137 | 0.39642155 | -0.0373243 | Prostate |
| ENSG00000071282 | 0.480582878 | 0.0704301 | Prostate |
| ENSG00000196220 | 0.487606476 | -0.0494445 | Prostate |
| ENSG00000156959 | 0.587391633 | 0.125928 | Prostate |
| ENSG00000125046 | 0.903475812 | 0.142393 | Prostate |
| ENSG00000227110 | 1.121558062 | 0.136261 | Prostate |
| ENSG00000180914 | 0.038532868 | -0.0191805 | sensory_neuron |
| ENSG00000227110 | 0.040281922 | -0.0134078 | sensory_neuron |
| ENSG00000168137 | 0.065777363 | -0.00748772 | sensory_neuron |
| ENSG00000206573 | 0.126605569 | 0.0196306 | sensory_neuron |
| ENSG00000196220 | 0.128632586 | -0.0249871 | sensory_neuron |
| ENSG00000156959 | 0.167905142 | 0.0269538 | sensory_neuron |
| ENSG00000071282 | 0.181933861 | 0.0723638 | sensory_neuron |
| ENSG00000231401 | 0.216295042 | -0.129657 | sensory_neuron |
| ENSG00000070950 | 0.245800787 | 0.0368791 | sensory_neuron |
| ENSG00000235830 | 0.28694481 | 0.0894199 | sensory_neuron |
| ENSG00000125046 | 0.374471485 | -0.210741 | sensory_neuron |
| ENSG00000134077 | 0.524633942 | -0.0780332 | sensory_neuron |
| ENSG00000206573 | 0.025035827 | -0.00168572 | skin |
| ENSG00000156959 | 0.04856582 | -0.00647138 | skin |
| ENSG00000070950 | 0.083412362 | -0.00520873 | skin |
| ENSG00000182533 | 0.133094453 | 0.0200218 | skin |
| ENSG00000196220 | 0.237707898 | -0.0183327 | skin |
| ENSG00000134077 | 0.351249787 | 0.0131752 | skin |
| ENSG00000180914 | 0.446489559 | -0.0433887 | skin |
| ENSG00000254485 | 0.648543843 | -0.0618846 | skin |
| ENSG00000071282 | 0.771923173 | -0.0392778 | skin |
| ENSG00000125046 | 0.88458588 | -0.0741008 | skin |
| ENSG00000227929 | 1.029000777 | 0.0659745 | skin |
| ENSG00000235830 | 1.528026571 | -0.132048 | skin |
| ENSG00000168137 | 1.812930729 | -0.0321258 | skin |
| ENSG00000134077 | 0.00119552 | 0.0001186 | Skin_Not_Sun_Exposed_Suprapubic |
| ENSG00000254485 | 0.178113257 | -0.0281211 | Skin_Not_Sun_Exposed_Suprapubic |
| ENSG00000196220 | 0.226451239 | -0.0188018 | Skin_Not_Sun_Exposed_Suprapubic |
| ENSG00000227110 | 0.234470024 | 0.0291492 | Skin_Not_Sun_Exposed_Suprapubic |
| ENSG00000070950 | 0.278058375 | -0.0229738 | Skin_Not_Sun_Exposed_Suprapubic |
| ENSG00000180914 | 0.296371637 | -0.038474 | Skin_Not_Sun_Exposed_Suprapubic |
| ENSG00000125046 | 0.299841656 | -0.034977 | Skin_Not_Sun_Exposed_Suprapubic |
| ENSG00000182533 | 0.487192909 | -0.0616068 | Skin_Not_Sun_Exposed_Suprapubic |
| ENSG00000071282 | 0.523730798 | 0.0301771 | Skin_Not_Sun_Exposed_Suprapubic |
| ENSG00000206573 | 0.709923075 | -0.0337732 | Skin_Not_Sun_Exposed_Suprapubic |
| ENSG00000156959 | 0.850673097 | 0.100362 | Skin_Not_Sun_Exposed_Suprapubic |
| ENSG00000168137 | 0.914841479 | 0.0324913 | Skin_Not_Sun_Exposed_Suprapubic |
| ENSG00000071282 | 0.070528962 | -0.00504287 | Skin_Sun_Exposed_Lower_leg |
| ENSG00000227110 | 0.090051572 | 0.0124349 | Skin_Sun_Exposed_Lower_leg |
| ENSG00000182533 | 0.154317297 | -0.0212494 | Skin_Sun_Exposed_Lower_leg |
| ENSG00000168137 | 0.172745132 | 0.00951335 | Skin_Sun_Exposed_Lower_leg |
| ENSG00000254485 | 0.203026937 | -0.0303493 | Skin_Sun_Exposed_Lower_leg |
| ENSG00000206573 | 0.326310186 | -0.0169894 | Skin_Sun_Exposed_Lower_leg |
| ENSG00000134077 | 0.370432412 | -0.0238214 | Skin_Sun_Exposed_Lower_leg |
| ENSG00000180914 | 0.595568277 | -0.0631933 | Skin_Sun_Exposed_Lower_leg |
| ENSG00000156959 | 0.633481789 | 0.0686614 | Skin_Sun_Exposed_Lower_leg |
| ENSG00000125046 | 0.678387073 | 0.0610238 | Skin_Sun_Exposed_Lower_leg |
| ENSG00000070950 | 0.747253736 | 0.0471903 | Skin_Sun_Exposed_Lower_leg |
| ENSG00000196220 | 0.964014241 | -0.0481427 | Skin_Sun_Exposed_Lower_leg |
| ENSG00000125046 | 0.059973369 | 0.00913023 | Small_Intestine_Terminal_Ileum |
| ENSG00000180914 | 0.065347048 | 0.0161449 | Small_Intestine_Terminal_Ileum |
| ENSG00000182533 | 0.119869244 | 0.0245457 | Small_Intestine_Terminal_Ileum |
| ENSG00000156959 | 0.146947018 | 0.0253083 | Small_Intestine_Terminal_Ileum |
| ENSG00000168137 | 0.160918089 | 0.0186396 | Small_Intestine_Terminal_Ileum |
| ENSG00000254485 | 0.212104032 | 0.0606295 | Small_Intestine_Terminal_Ileum |
| ENSG00000134077 | 0.35268257 | 0.0490299 | Small_Intestine_Terminal_Ileum |
| ENSG00000206573 | 0.396052763 | 0.0432193 | Small_Intestine_Terminal_Ileum |
| ENSG00000070950 | 0.399537891 | -0.0571835 | Small_Intestine_Terminal_Ileum |
| ENSG00000196220 | 0.499388964 | -0.0873785 | Small_Intestine_Terminal_Ileum |
| ENSG00000071282 | 0.564442287 | 0.06363 | Small_Intestine_Terminal_Ileum |
| ENSG00000206573 | 0.013167827 | 0.00188952 | Spleen |
| ENSG00000125046 | 0.039866134 | 0.00901788 | Spleen |
| ENSG00000196220 | 0.123944473 | -0.0255911 | Spleen |
| ENSG00000180914 | 0.149070249 | 0.0328533 | Spleen |
| ENSG00000227110 | 0.186523762 | -0.040253 | Spleen |
| ENSG00000182533 | 0.191872112 | -0.0409211 | Spleen |
| ENSG00000134077 | 0.199122839 | -0.0318713 | Spleen |
| ENSG00000070950 | 0.355229661 | -0.051155 | Spleen |
| ENSG00000254485 | 0.675647569 | -0.128997 | Spleen |
| ENSG00000071282 | 0.779314109 | 0.0703162 | Spleen |
| ENSG00000168137 | 0.829277825 | -0.059627 | Spleen |
| ENSG00000254485 | 0.028602603 | -0.00577207 | Stomach |
| ENSG00000182533 | 0.045414051 | 0.00962093 | Stomach |
| ENSG00000196220 | 0.052841844 | -0.00678541 | Stomach |
| ENSG00000227110 | 0.063100389 | -0.0128198 | Stomach |
| ENSG00000156959 | 0.085368643 | -0.0137844 | Stomach |
| ENSG00000180914 | 0.265396967 | 0.0410407 | Stomach |
| ENSG00000134077 | 0.329625177 | -0.0307581 | Stomach |
| ENSG00000168137 | 0.514798335 | -0.0338774 | Stomach |
| ENSG00000071282 | 0.60149404 | 0.0481303 | Stomach |
| ENSG00000125046 | 0.696484281 | 0.0849474 | Stomach |
| ENSG00000070950 | 0.774339289 | 0.0734063 | Stomach |
| ENSG00000206573 | 1.490110948 | 0.0866438 | Stomach |
| ENSG00000070950 | 0.001771185 | -7.83E-05 | T-cell |
| ENSG00000168137 | 0.052517754 | -0.00157022 | T-cell |
| ENSG00000206573 | 0.075184381 | -0.00661418 | T-cell |
| ENSG00000228723 | 0.163133039 | 0.0353288 | T-cell |
| ENSG00000235830 | 0.186527098 | -0.0260255 | T-cell |
| ENSG00000196220 | 0.268048803 | -0.0280408 | T-cell |
| ENSG00000134077 | 0.361452841 | 0.0122619 | T-cell |
| ENSG00000224808 | 0.446114547 | 0.0657114 | T-cell |
| ENSG00000227929 | 0.46005618 | 0.0766875 | T-cell |
| ENSG00000254485 | 0.721081855 | -0.128239 | T-cell |
| ENSG00000071282 | 0.953477522 | -0.193547 | T-cell |
| ENSG00000180914 | 1.28766435 | 0.124309 | T-cell |
| ENSG00000180914 | 0.018444585 | -0.00371143 | Testis |
| ENSG00000206573 | 0.046952478 | 0.00505733 | Testis |
| ENSG00000228723 | 0.091832507 | -0.0228087 | Testis |
| ENSG00000254485 | 0.110486526 | 0.0199985 | Testis |
| ENSG00000070950 | 0.123673022 | 0.0153825 | Testis |
| ENSG00000224808 | 0.139652409 | 0.0296248 | Testis |
| ENSG00000227929 | 0.16921123 | 0.0179747 | Testis |
| ENSG00000228351 | 0.171623067 | -0.0341635 | Testis |
| ENSG00000270207 | 0.220156132 | 0.0486956 | Testis |
| ENSG00000134077 | 0.342328536 | -0.0193638 | Testis |
| ENSG00000231401 | 0.363951635 | 0.0634005 | Testis |
| ENSG00000215160 | 0.53117875 | -0.0718448 | Testis |
| ENSG00000168137 | 0.56522677 | 0.0239774 | Testis |
| ENSG00000227110 | 0.605663913 | 0.104329 | Testis |
| ENSG00000182533 | 0.723076573 | 0.0817759 | Testis |
| ENSG00000156959 | 0.961809179 | 0.0929923 | Testis |
| ENSG00000125046 | 1.204186 | -0.0482902 | Testis |
| ENSG00000071282 | 1.312877879 | 0.0696437 | Testis |
| ENSG00000196220 | 1.458392054 | 0.0974795 | Testis |
| ENSG00000196220 | 0.039833287 | -0.0122211 | Tfh_memory |
| ENSG00000168137 | 0.182614467 | 0.00463949 | Tfh_memory |
| ENSG00000206573 | 0.476465531 | 0.0336708 | Tfh_memory |
| ENSG00000070950 | 0.638209857 | -0.0287586 | Tfh_memory |
| ENSG00000134077 | 0.648654063 | -0.0307755 | Tfh_memory |
| ENSG00000180914 | 1.934054482 | -0.343658 | Tfh_memory |
| ENSG00000134077 | 0.083201385 | 0.00575477 | Th1_memory |
| ENSG00000070950 | 0.1777384 | 0.0114275 | Th1_memory |
| ENSG00000168137 | 0.178374473 | -0.00633214 | Th1_memory |
| ENSG00000180914 | 0.486096863 | -0.127193 | Th1_memory |
| ENSG00000206573 | 1.507549733 | 0.0774395 | Th1_memory |
| ENSG00000196220 | 2.078777193 | 0.235424 | Th1_memory |
| ENSG00000168137 | 0.02669246 | -0.000902209 | Th1-17_memory |
| ENSG00000134077 | 0.154249767 | -0.0086399 | Th1-17_memory |
| ENSG00000070950 | 0.539780426 | 0.0203795 | Th1-17_memory |
| ENSG00000180914 | 0.65569027 | -0.144113 | Th1-17_memory |
| ENSG00000206573 | 0.944828161 | 0.0497316 | Th1-17_memory |
| ENSG00000134077 | 0.114778478 | 0.00760282 | Th17_memory |
| ENSG00000070950 | 0.200132413 | 0.00846864 | Th17_memory |
| ENSG00000071282 | 0.31692413 | 0.09139 | Th17_memory |
| ENSG00000168137 | 0.660107532 | 0.0157906 | Th17_memory |
| ENSG00000180914 | 1.460858636 | -0.230574 | Th17_memory |
| ENSG00000206573 | 2.204358389 | 0.0924117 | Th17_memory |
| ENSG00000134077 | 0.032609765 | 0.00236938 | Th2_memory |
| ENSG00000071282 | 0.24207585 | 0.0916211 | Th2_memory |
| ENSG00000206573 | 0.342868583 | 0.027264 | Th2_memory |
| ENSG00000196220 | 0.570165389 | -0.125178 | Th2_memory |
| ENSG00000070950 | 0.620525875 | 0.0294811 | Th2_memory |
| ENSG00000168137 | 0.624475383 | -0.0179966 | Th2_memory |
| ENSG00000180914 | 0.922043743 | -0.223543 | Th2_memory |
| ENSG00000070950 | 0.053655339 | 0.00518769 | Thyroid |
| ENSG00000125046 | 0.05518877 | 0.00692483 | Thyroid |
| ENSG00000235830 | 0.086658302 | 0.01211 | Thyroid |
| ENSG00000214041 | 0.102197577 | -0.0143566 | Thyroid |
| ENSG00000180914 | 0.155625352 | -0.0206551 | Thyroid |
| ENSG00000196220 | 0.23701926 | 0.020547 | Thyroid |
| ENSG00000182533 | 0.305290407 | -0.0369302 | Thyroid |
| ENSG00000156959 | 0.329663214 | 0.0452971 | Thyroid |
| ENSG00000254485 | 0.341917022 | -0.0357836 | Thyroid |
| ENSG00000206573 | 0.600314167 | -0.0293514 | Thyroid |
| ENSG00000134077 | 0.771717728 | -0.0356636 | Thyroid |
| ENSG00000071282 | 1.375903628 | 0.0846869 | Thyroid |
| ENSG00000168137 | 1.923410524 | 0.060304 | Thyroid |
| ENSG00000227110 | 3.150424588 | 0.183864 | Thyroid |
| ENSG00000071282 | 0.077565726 | -0.0112977 | Treg_memory |
| ENSG00000134077 | 0.153059502 | 0.00963089 | Treg_memory |
| ENSG00000196220 | 0.301634085 | -0.0367985 | Treg_memory |
| ENSG00000168137 | 0.308964505 | 0.0101777 | Treg_memory |
| ENSG00000070950 | 0.817759443 | -0.0339362 | Treg_memory |
| ENSG00000206573 | 1.379110572 | 0.0655627 | Treg_memory |
| ENSG00000180914 | 2.18195499 | -0.354189 | Treg_memory |
| ENSG00000070950 | 0.000835082 | -5.44E-05 | Treg_naive |
| ENSG00000206573 | 0.369690123 | 0.0307634 | Treg_naive |
| ENSG00000180914 | 0.39053259 | -0.150258 | Treg_naive |
| ENSG00000168137 | 0.551292135 | -0.0160973 | Treg_naive |
| ENSG00000196220 | 0.731099235 | -0.109982 | Treg_naive |
| ENSG00000134077 | 0.816067118 | 0.0395257 | Treg_naive |
| ENSG00000254485 | 0.909227167 | -0.274741 | Treg_naive |
| ENSG00000071282 | 1.480732621 | -0.343487 | Treg_naive |
| ENSG00000125046 | 0.069452355 | 0.0200512 | Uterus |
| ENSG00000071282 | 0.144873522 | -0.0252251 | Uterus |
| ENSG00000196220 | 0.158334003 | 0.0336958 | Uterus |
| ENSG00000227110 | 0.186736676 | 0.0516484 | Uterus |
| ENSG00000156959 | 0.205258979 | 0.0638798 | Uterus |
| ENSG00000206573 | 0.258909885 | 0.0426231 | Uterus |
| ENSG00000182533 | 0.261921378 | -0.0704208 | Uterus |
| ENSG00000070950 | 0.449931937 | -0.0961824 | Uterus |
| ENSG00000134077 | 0.455540027 | -0.0636076 | Uterus |
| ENSG00000180914 | 0.489916338 | 0.122624 | Uterus |
| ENSG00000168137 | 0.518832655 | -0.0555581 | Uterus |
| ENSG00000180914 | 0.018802711 | -0.00635992 | Vagina |
| ENSG00000071282 | 0.020947204 | -0.00386746 | Vagina |
| ENSG00000168137 | 0.039802348 | 0.0062534 | Vagina |
| ENSG00000182533 | 0.09882458 | 0.0298526 | Vagina |
| ENSG00000227110 | 0.101161873 | -0.0255221 | Vagina |
| ENSG00000070950 | 0.141735375 | 0.036397 | Vagina |
| ENSG00000206573 | 0.277372658 | 0.0585768 | Vagina |
| ENSG00000134077 | 0.484224148 | 0.0629231 | Vagina |
| ENSG00000125046 | 0.525524403 | -0.121508 | Vagina |
| ENSG00000156959 | 0.692771467 | 0.168654 | Vagina |
| ENSG00000196220 | 0.830707725 | -0.140714 | Vagina |
| ENSG00000071282 | 0.014040753 | 0.00136251 | Whole_Blood |
| ENSG00000206573 | 0.123087959 | 0.00841213 | Whole_Blood |
| ENSG00000168137 | 0.569700757 | -0.015669 | Whole_Blood |
| ENSG00000070950 | 0.575149386 | 0.0344457 | Whole_Blood |
| ENSG00000180914 | 0.678436778 | -0.0704005 | Whole_Blood |
| ENSG00000196220 | 0.787098927 | 0.0624084 | Whole_Blood |
| ENSG00000134077 | 1.533572826 | -0.0700233 | Whole_Blood |
